# Plk1 relieves centriole block to reduplication by promoting daughter centriole maturation

**DOI:** 10.1038/ncomms9077

**Published:** 2015-08-21

**Authors:** Anil Shukla, Dong Kong, Meena Sharma, Valentin Magidson, Jadranka Loncarek

**Affiliations:** 1 Laboratory of Protein Dynamics and Signaling, 1050 Boyles Street, NIH/NCI/CCR, Frederick, Maryland 21702, USA; 2 Optical Microscopy and Analysis Laboratory, Leidos Biomedical Res Inc., Frederick National Laboratory for Cancer Research, Frederick, Maryland 21702, USA

## Abstract

Centrosome overduplication promotes mitotic abnormalities, invasion and tumorigenesis. Cells regulate the number of centrosomes by limiting centriole duplication to once per cell cycle. The orthogonal orientation between a mother and a daughter centriole, established at the time of centriole duplication, is thought to block further duplication of the mother centriole. Loss of orthogonal orientation (disengagement) between two centrioles during anaphase is considered a licensing event for the next round of centriole duplication. Disengagement requires the activity of Polo-like kinase 1 (Plk1), but how Plk1 drives this process is not clear. Here we employ correlative live/electron microscopy and demonstrate that Plk1 induces maturation and distancing of the daughter centriole, allowing reduplication of the mother centriole even if the original daughter centriole is still orthogonal to it. We find that mother centrioles can undergo reduplication when original daughter centrioles are only ∼80 nm apart, which is the distance centrioles normally reach during prophase.

## Abstract (web summary)

The orthogonal orientation between centrioles is thought to prevent their reduplication. Shukla *et al.* show that Polo-like kinase 1-dependent daughter centriole maturation, reflected in increasing inter-centriolar distance, allows centriole reduplication prior to loss of orthogonal orientation.

## Graphical abstract

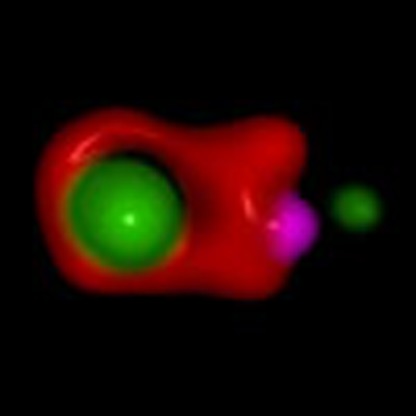

A typical centrosome consists of one unduplicated or duplicated centriole^1^, surrounded by pericentriolar material (PCM), which is responsible for most centrosomal functions. The number of centrosomes is determined by the number of (mature) centrioles capable of organizing the PCM, which is otherwise structurally unstable^2^. A cell contains two mature (mother) centrioles, which duplicate in early S forming a new (daughter) centriole in an orthogonal configuration at their proximal end. Daughter centrioles are initially immature, but gain the ability to organize a PCM in the next cell cycle. Orthogonal configuration of mother and daughter centriole pairs is thought to block the mother centriole from forming additional daughter centrioles during the same cell cycle. Disengagement, defined as a loss of orthogonal orientation between centrioles, is thought to occur after anaphase and is considered a licensing event for the next round of centriole duplication^3^,^4^. However, the nature of the block to reduplication and mechanism(s) of centriole disengagement are unknown.

Expression of either wild-type Polo-like kinase 1 (Plk1) or constitutively active Plk1T210D (Plk1TD)^5^, or arresting cells in G2 with uninhibited endogenous Plk1 (ref. 6), promotes disengagement of mother and daughter centrioles and, in turn, allows their reduplication. How engagement between the centrioles inhibits formation of new daughter centrioles remains a long-standing question.

Ablation of daughter centrioles from engaged mother–daughter centriole pairs by a laser microbeam^7^ primes mother centrioles in S-phase-arrested HeLa cells for a new round of duplication. Thus, the presence of a daughter centriole within the PCM attenuates the duplication ability of mother centrioles. Much effort has been put forward in recent years to identify molecular mechanisms responsible for resolving the orthogonal orientation of mother–daughter centrioles within the centriole pairs. Centriole disengagement in vertebrates requires Plk1 activity, and is possibly facilitated by the activity of Separase^8^, a protease that cleaves Cohesin at the end of mitosis to allow separation of sister chromatids. However, how Plk1 drives centriole disengagement is not clear.

In this manuscript, we employ correlative live-cell electron microscopy to explore Plk1-dependent intra-centrosomal ultrastructural rearrangements leading to the relief of centriole block to reduplication. Our analysis reveals that centriole block to reduplication relies on close spatial association of mother and daughter centrioles, and not on their orthogonal orientation.

We find that Plk1-dependent maturation of daughter centrioles promotes their distancing from mother centrioles, leading to loss of the centriole block to reduplication.

We propose that centriole disorientation following centriole distancing is a facultative event, the dynamics of which may vary depending on specific conditions at the time of centriole distancing. We found that mother centrioles can reduplicate even when the original daughter centrioles are only ∼80 nm apart. We also show that mother–daughter centriole distance increases during the cell cycle reaching the distance of ∼80 nm at the time of prophase. These data point towards an exciting possibility that centriole block to reduplication in cycling human cells may already be lost upon mitotic entry, and not after metaphase to anaphase transition, as currently believed.

## Results

### Centriole block to reduplication is short ranged

To describe the earliest ultrastructural changes that occur within the centrosomes during centriole disengagement, we employed correlative live and electron microscopy. We used cells constitutively expressing Centrin1 fused with green fluorescent protein (C1–GFP) to label distal parts of centrioles. C1–GFP incorporates into the distal lumen of centrioles from the earliest stages of their formation^9^. Cells also expressed a doxycycline (dox)-inducible constitutively active Plk1T210D mutant (Plk1TD) fused with red fluorescent protein (RFP). The expression of wild-type Plk1 or Plk1TD (but not of a kinase-dead mutant) leads to centriole disengagement and reduplication in S-phase-arrested or cycling human cells^5^.

**Figure 1:**
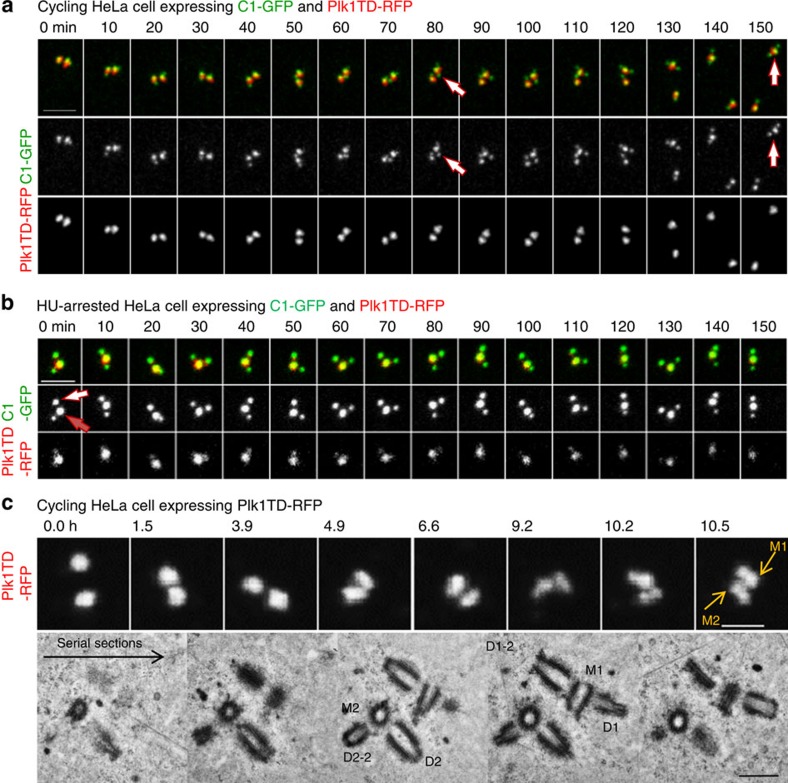
Centriole block to reduplication is short ranged. (**a–c**) Stills from time-lapse recordings showing centrioles in reduplication. (**a**) Two Plk1TD–RFP signals indicate two centrosomes. Each Plk1TD–RFP signal is associated with one brighter C1–GFP signal (mother centriole) and one or two dimmer C1–GFP signals (daughter centrioles). The two centrosomes separate during imaging, revealing that one mother centriole is associated with two daughter centrioles (80-min time frame, indicated by an arrow). The mother moves in the cytoplasm associated with both daughter centrioles (150 min, indicated by an arrow). (**b**) A mother centriole (red arrow) in reduplication is still closely associated with an older daughter centriole (white arrow). Scale bars, 2 μm (**a,b**). (**c**) Correlative light and electron analysis of centrioles in reduplication. Two centrosomes in a Plk1TD–RFP-expressing cell were first followed by time lapse. Initially two symmetrical signals are visible. Later, the signals became more elongated, indicating that the daughter centriole matured and accumulated Plk1TD–RFP. Scale bar, 2 μm. At that point, the cell was fixed and the same centrosomes were analysed by electron microscopy. Electron microscopy revealed that both mother centrioles (M1 and M2) reduplicated and were associated with shorter daughter centrioles (D1–2 and D2–2) at the distance of ∼65 nm, and with the original longer daughter centriole (D1 and D2) in orthogonal or almost orthogonal orientation, but at a distance greater than 80 nm.

We followed the behaviour of centrioles after Plk1TD–RFP expression by long-term time-lapse microscopy. This analysis revealed that mother centrioles can initiate the formation of a new daughter centriole while still in close association with the original daughter centriole (Fig. 1a). Reduplicating mother centrioles were sometimes associated with the original daughter even several hours, after the formation of a new daughter was first detected. The original daughters eventually separated, which could occur at any phase of the cell cycle. The same behaviour was observed during centriole reduplication in S-phase-arrested cells expressing Plk1TD–RFP (Fig. 1b) or in G2-arrested cells. A correlative light/electron microscopy (CLEM) analysis of such mother centrioles in reduplication surprisingly revealed that the original daughter centrioles could still be orthogonal or almost orthogonal to the mother centrioles, even when the newly formed daughter centrioles are already substantially elongated (Fig. 1c). Therefore, opposite of what is currently believed, these data show that centriole disorientation (thus disengagement as it is currently defined), may not be required for loss of centriole block to reduplication in human cells.

We noticed, however, that original daughters were positioned slightly farther (∼90 nm) from mother centrioles than younger daughters (∼65 nm). Thus, we hypothesized that centriole block to reduplication relies on maintenance of a critical minimal distance between two centrioles, and proceeded to analyse the earliest ultra-structural and biochemical changes that occur within the centrosome leading to the loss of centriole block to reduplication.

### Centriole configuration can be determined by light microscopy

**Figure 2:**
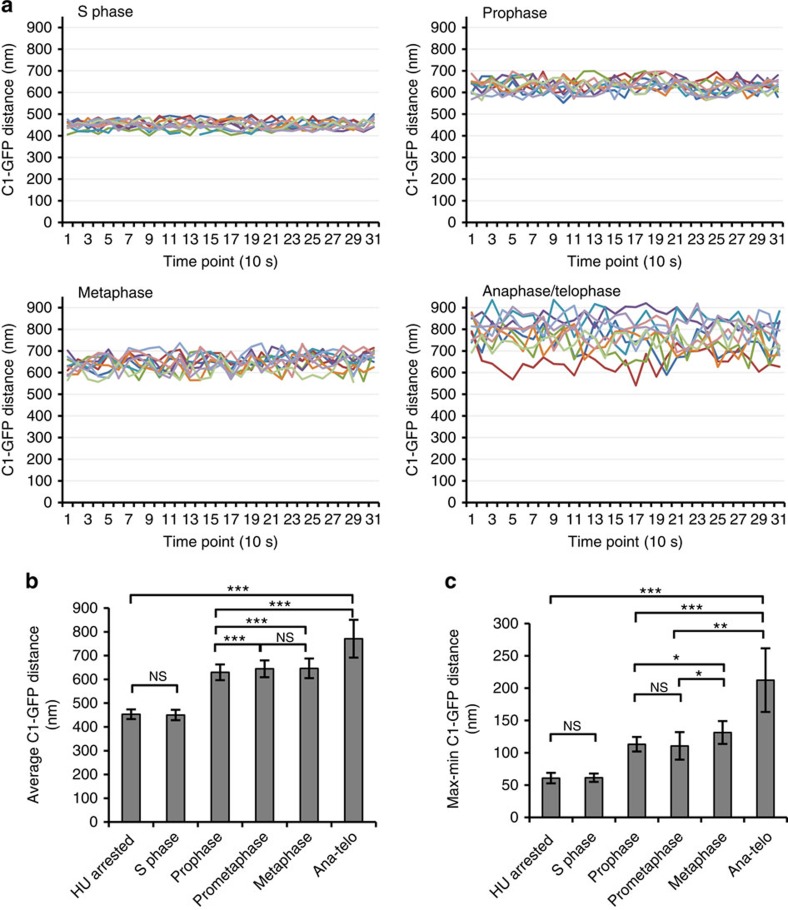
The configuration of a centriole pair can be determined by light microscopy. (**a**) Analysis of centriole movement within C1–GFP-expressing cells. Mother and daughter centriole pairs were imaged during 5 min with 10 s resolution. Distances between mother and daughter-associated C1–GFP signals were calculated and plotted. In cycling S-phase cells, the average distance between C1–GFP signals during the imaging period is 450 nm, with small oscillations. In prophase and metaphase cells, the C1–GFP distance is ∼650 nm, and oscillations between the centrioles increased twofold with respect to the S phase. Centrioles in anaphase and telophase move at distances that are larger than 700 nm and with large oscillations between the time points, indicating that the two centrioles lost association. Each line on the graph represents one mother–daughter centriole pair. (**b**) Histogram represents the average mother-to-daughter centriole C1–GFP distance from all measured centriole pairs for the indicated phase of the cell cycle, error bars are the s.d. (*n*=10 centriole pairs, 31 time points per pair). (**c**) Histogram represents average differences between maximal and minimal C1–GFP values from the data set presented in **b** (average from 10 time-lapse recordings, 31 time points per recording), error bars are the s.d. The statistical significance between the data sets was determined by a two-tailed *t*-test using Microsoft excel. NS, not statistically different, *P≤0.05, **P≤0.01, ***P≤0.001.

We first established that live-cell microscopy can be used to predict centriole configuration within the centrosome. Mother- and daughter-associated C1–GFP was used as a marker for distal parts of the two centrioles. Z-sections spanning both centrioles were recorded during 5 min with 10-s time resolution. Three-dimensional (3D) coordinates of mother and daughter C1–GFP signals were extracted for each time point, the distances between C1–GFP signals were calculated and plotted over time (Fig. 2a; Supplementary Fig. 8).

In cycling S-phase and S-phase-arrested cells, the average distance between C1–GFP signals during the imaging period was ∼450 nm, with only small differences between maximal and minimal measured values; thus, centriole pairs tumble with small oscillations (Fig. 2). In prophase and metaphase cells, the C1–GFP distance increased to ∼650 nm reflecting elongation of the daughter centrioles that normally occurs at the latest stages of the cell cycle. However, oscillations between the centrioles increased twofold with respect to the centrioles in the S phase, meaning that the centrioles in prophase and metaphase were already losing rigid configuration they maintained throughout the S phase. Centrioles in anaphase and telophase tumbled at highly oscillating distances larger than 700 nm, indicating that they lost their rigid configuration.

Interestingly, analysis of C1–GFP distances in S-phase-arrested Plk1TD-expressing cells undergoing centriole reduplication (as illustrated in Fig. 1b) revealed that the average C1–GFP distance between the mother and older daughter centrioles was only 670 nm (Supplementary Fig. 1). In addition, while newly formed daughter centrioles moved in synchrony with the mother centrioles, the original daughter centrioles moved at the oscillating distance similar to that measured during cycling prometaphase and metaphase cells.

Same-cell CLEM analysis further confirmed that the values between C1–GFP signals could be used to determine the distance between the distal ends of the centrioles (Supplementary Fig. 2a). It also revealed that in the HeLa cells used in this study, C1–GFP is positioned ∼30 nm from the distal end of the centrioles.

### Centrioles distanced to 80 nm begin to disorient

We next used light microscopy to identify centriole pairs that are likely undergoing disengagement, and to correlate ultrastructural changes within the centrosome with biochemical features. For that, we used cells arrested in G2 by Cdk1 inhibitor RO-3306 (ref. 10). Several hours after reaching G2, centrioles in these cells disengage and reduplicate^6^. Thus, they can be used to study both events during interphase. First, by measuring the length of the centrioles from electron micrographs, we determined that the average mother and daughter centriole length in G2-arrested cells is 484±49 (*n*=12) and 408±43 (*n*=11) nm, respectively. These values allowed us to further calculate maximal theoretical C1–GFP distance of two orthogonally oriented centrioles during G2 arrest, which we found to be ∼650 nm (Supplementary Fig. 2b). We further used this value as a criterion to identify centriole pairs that are likely undergoing disengagement, as any distance greater than 650 nm would indicate that centrioles either disoriented or moved apart.

**Figure 3:**
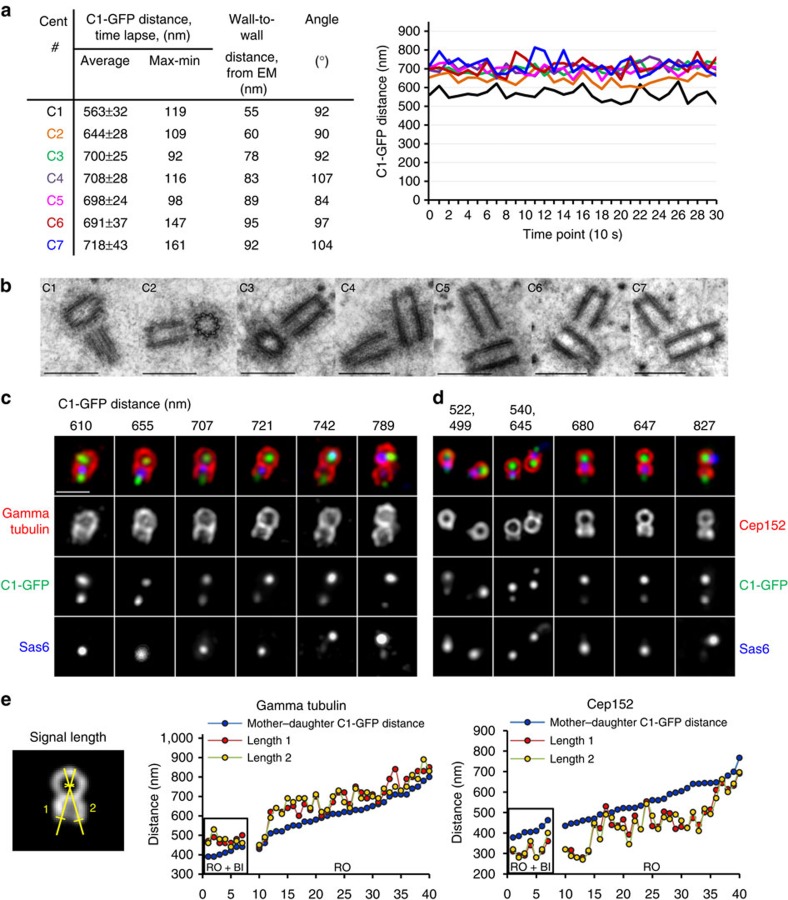
Analysis of ultra-structural changes within the centrosome during the earliest stages of disengagement. (**a**,**b**) Centrioles of C1–GFP-expressing cells arrested in G2 by Cdk1 inhibitor RO-3306 (RO) were first analysed by 5 min time lapse to determine the pattern of centriole movement (expressed as the average distance between the mother and daughter C1–GFP signals). The same centrioles were then fixed and analysed by electron microscopy. Mother–daughter centriole pairs with average C1–GFP distance ≤700 nm were found in orthogonal orientation and at the wall-to-wall distance <80 nm. Daughter centrioles ≥80 nm from the wall of the mother centrioles were found disoriented. Scale bars, 500 nm. (**c**,**d**) Daughter centrioles in the centrosomes with C1–GFP distances ≤700 nm are associated with a substantial amount of PCM (as evidenced by a gamma tubulin signal) and with licensing factor Cep152. Accumulation of Cep152 precedes the degradation of Sas6, separation of two PCMs and loss of orthogonal orientation between the centrioles. Scale bar, 1 μm. (**e**) Quantification of daughter centriole-associated gamma tubulin and Cep152. Only centrioles with mother centriole perpendicular to the coverslip were measured. For each centrosome, two signal lengths were measured from the centre of a gamma tubulin or Cep152 toroid organized by the mother centriole, until the end of the signal associated with the daughter centriole (determined as a signal half maximum). Centrioles in cells arrested in G2 and treated with Plk1 inhibitor BI2536 (RO+BI) were measured as a control.

In the population of G2-arrested cells, three categories of mother–daughter centriole pairs can be distinguished. In the first category, the average C1–GFP distance was ≤650 nm (Fig. 3a,c). Both centrioles in such centrosomes were still embedded in one cloud of gamma tubulin. In the second category, C1–GFP distance exceeded 750 nm, and two adjacent, but independent PCM signals were resolvable, indicating that two centrioles were independent. Finally, in the third category, the PCM surrounding mother–daughter pairs was elongated but not yet clearly resolvable as two discrete entities, and the centrioles were at the average C1–GFP distance of 650–750 nm. This range was above theoretical limit of 650 nm, suggesting that the centrioles might be already disoriented or at larger distances, and we analysed such centrioles by CLEM.

Indeed correlative analysis of such centrioles (Fig. 3a,b; Supplementary Fig. 3) revealed that in the centrosomes with C1–GFP distances between 600 and 800 nm, the daughters can be at various distances from the wall of the mother centrioles. Daughter centrioles whose proximal parts were found to be closer than 80 nm to the wall of the mother centrioles were found orthogonal or near orthogonal, whereas those at greater distances mostly lost orthogonal orientation, and/or were shifted along the wall of the mother centriole. Therefore, we concluded that centrioles begin to disorient after they reach a wall-to-wall distance of ∼80 nm.

### Centriole distancing relieves the reduplication block

During the unperturbed cell cycle, daughter centrioles do not organize their own PCM before the end of mitosis^11^. However, in conditions that allow centriole reduplication, even in the centrosomes with mother–daughter C1–GFP distances below 650 nm, daughter centrioles were already associated with a substantial amount of gamma tubulin, and, interestingly, with Cep152/Asterless (Fig. 3c–e). Cep152 normally accumulates at the proximal portions of disengaged daughter centrioles during G1, and is regarded as a licensing factor because it recruits the centriole duplication kinase Plk4 to the centrioles^12^,^13^,^14^,^15^. Gamma tubulin and Cep152-positive daughter centrioles were also still associated with Sas6. Sas6 is one of the first proteins accumulating to the proximal ends of the daughter centriole formation during early S phase, and is present there until it is normally degraded in an APC/C^cdh1^-dependent fashion at the end of mitosis^16^,^17^. Therefore, the association of interphase levels of Sas6 with Cep152-positive daughter centrioles means that Sas6 degradation is not required for Cep152 accumulation on daughter centrioles. Cep152-positive daughter centrioles could still be found orthogonal to the mothers, even if a mother centriole was already in reduplication and associated with a new Sas6 signal; thus, block to reduplication was relieved in such centrosomes.

In conclusion, these data show that daughter centrioles begin gradual accumulation of PCM while still orthogonal to the mother centriole.

### Mother-to-daughter distance increases during cell cycle

**Figure 4:**
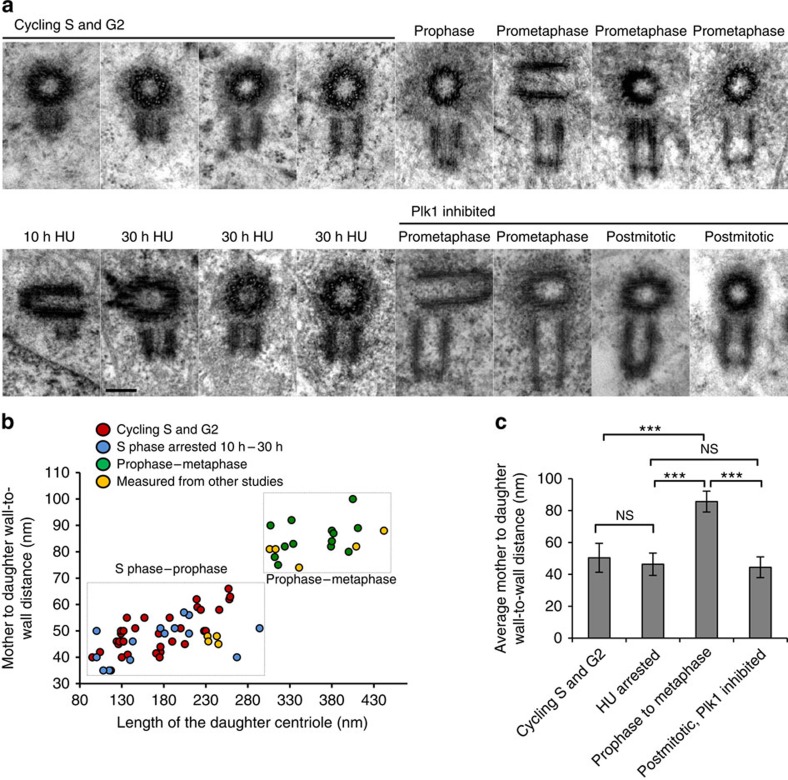
The distance between mother and daughter centrioles increases during the cell cycle in a Plk1-dependent manner. (**a**) Electron micrographs of duplicated centrioles with the daughter centrioles of various lengths. Daughter centrioles from cycling S and G2-phase-arrested cells and from cells arrested in the S phase by hydroxyurea (HU) are positioned closer to the walls of the mother centrioles than the daughter centrioles in prometaphase and metaphase cells. After inhibition of endogenous Plk1 for 4 h before mitotic entry, daughter centrioles remain close to the mother centrioles during prometaphase. The same is true for the centrioles in post-mitotic cells, if Plk1 is inhibited 7 h before mitotic entry. Scale bar, 200 nm. (**b,c**) Graph showing the correlation between mother–daughter centriole wall-to-wall distance and daughter centriole length. Wall-to-wall distance and the length of daughter centrioles were measured from electron micrographs. Points marked in yellow were measure from electron micrographs from refs 1, 18, 19, 20. Each dot on the graph represents one centriole pair. (**c**) Histogram represents average wall-to-wall centriole distance under various experimental conditions. Error bars are the s.d. The statistical significance between the data sets was determined by a two-tailed *t*-test using Microsoft excel. NS, not statistically different, ***P≤0.001.

To determine when during an unperturbed cell cycle mother–daughter centriole pairs separate by a distance of 80 nm, which is sufficient for centriole disorientation, we analysed cycling HeLa cells from early S to telophase using electron micrographs. The results are presented in Fig. 4 as a correlation between daughter centriole length and its distance from the wall of the mother centriole. Daughter centrioles shorter than 150 nm were found in close association with the mother centriole, with an average distance of 45±5 nm (*n*=14) from the wall of the mother centrioles. Daughter centrioles in the S phase, between 150 and 300 nm in length, were found 53±9 nm (*n*=22) from the wall of the mother centriole. The centriole distance in the S-phase-arrested cells, treated with hydroxyurea (HU) was found to be 46±7 nm (*n*=14), which was not significantly different to that measured for cycling cells in the S phase (Fig. 4c). However, in prophase and prometaphase cells, average distance between the centrioles was increased to 86±6 nm (*n*=12). Therefore, by the time a cell reaches prophase, proximal ends of the daughter centrioles are already ∼40 nm away from their initial assembly site (Fig. 4b).

We additionally searched the published literature^1^,^18^,^19^,^20^ for electron micrographs of duplicated centrioles from various cycling cells, and correlated the lengths of these daughter centrioles and their distance from their mothers. Interestingly, irrespective of the cell type (HeLa, PE, CHO and L929), all measured centriole pairs followed the trend measured for HeLa cells (Fig. 4, yellow marks in the graph). Therefore, distancing of the daughter centriole during the cell cycle seems to be a universal phenomenon for the mammalian centrosome.

### Centriole distancing is Plk1 dependent

We next reasoned that centriole distancing should be dependent on centrosome-associated Plk1 activity (Fig. 4a,c). Indeed, in cells arrested in prometaphase by inhibition of Plk1 for 4 h before mitotic entry, the average centriole wall-to-wall distance was only 55 nm (*n*=3). Accordingly, the wall-to-wall distances measured in post-mitotic cells, obtained after inhibiting Plk1 for 7 h prior to mitotic entry was 44±6 nm (*n*=9), and not different from that measured in the S-phase cells. Therefore, we concluded that centriole distancing beyond ∼55 nm requires Plk1 activity.

### Centrioles can reduplicate in post-mitotic cells

If in cycling cells centriole block to reduplication is already relieved at the time of mitotic entry, then it should be independent of mitotic events associated with metaphase to anaphase transition. Indeed, it has been shown previously that inhibition of Plk1 several hours before mitotic entry, but not after cells already committed to mitosis, inhibits centriole duplication in the next cell cycle^8^. One plausible explanation for this phenotype is that the centrioles in cells committed to mitosis have already reached the critical distance and lost the block to reduplication. To test this prediction, we inhibited endogenous Plk1 in the population of cycling cells and collected prometaphase-arrested cells that entered mitosis within 1 or 4 h after Plk1 inhibition. We reasoned that the centrioles were already distanced in cells closer to mitosis, but not those earlier in G2. We next promoted mitotic exit by addition of Cdk1 inhibitor, fixed post-mitotic cells 1.5 h later and analysed their centrioles.

**Figure 5:**
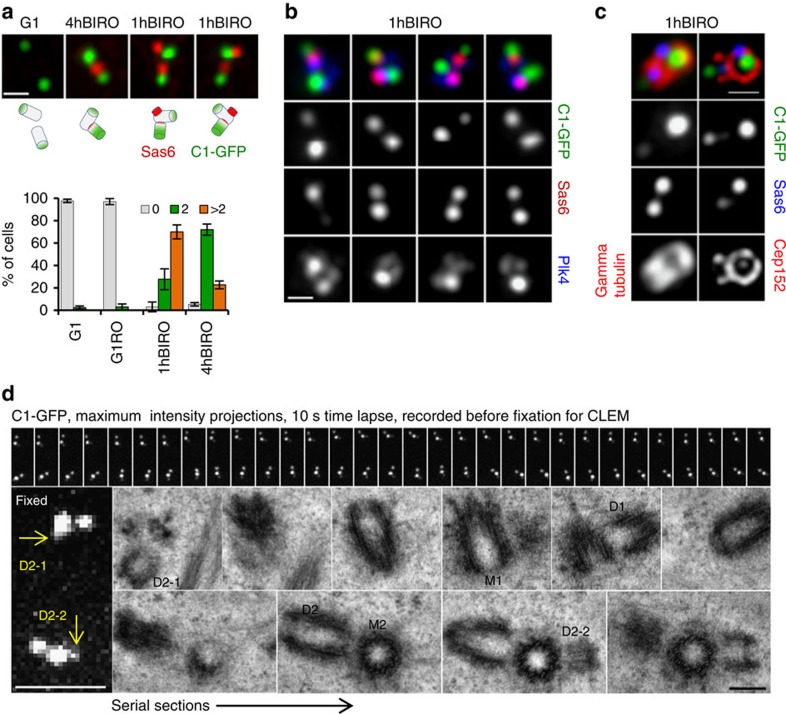
Plk1 inhibition in late G2 allows centriole duplication in post-mitotic cells. (**a**) Analysis of centrosome-associated Sas6 signal by immunofluorescence. In control G1 cells, mother and daughter centrioles are separated and Sas6 is absent. In the 4hBIRO sample, mother and daughter centrioles are adjacent, and associated with one Sas6. 1hBIRO sample contains two adjacent centrioles associated with two Sas6 signals, indicative that mother centriole is reduplicating. Schematics of putative centriole configurations are provided to facilitate understanding. Histogram represents quantification of the cells with 0, 2 or >2 Sas6 signals. Average value and the s.d. are presented. *n*=300, from three independent experiments. (**b**) In 1hBIRO cells, one of the Sas6 signals is associated with a mother centriole (brighter C1–GFP signal) and is co-localized with Plk4 signal. (**c**) In centrosomes with two Sas6 signals, the older daughter centriole is organizing gamma tubulin and Cep152. Its Sas6 signal is positioned outside the Cep152 toroid organized by the mother centriole. The centre of nascent Sas6 signal co-localizes with the mother's Cep152 toroid. Scale bars, 400 nm (**a**–**c**). (**d**) Correlative time-lapse and electron microscopy analysis of a post-mitotic cell from 1hBIRO samples. A 5-min-long time-lapse recording with 10 s resolution reveals weak C1–GFP signals (arrows) associated with the mother centrioles, in addition to brighter C1–GFP signals. Electron microscopy further confirmed that the weak C1–GFP signals correspond to the nascent daughter centrioles (D1–2 and D2–2) positioned close to the wall of the mother centrioles. Original daughter centrioles (D1 and D2) were still in an orthogonal orientation to the mother centriole. Scale bars, 200 nm.

Treatment of mitotic cells with Cdk1 inhibitor promotes rapid exit into G1 (ref. 10). Chromatin in these cells decondenses, centrioles disengage and continue to duplicate as cells progress into the next S phase^8^. Accordingly, in control G1 cells, and in post-mitotic cells treated only with Cdk1 inhibitor (RO), the centrioles were separated and Sas6 was absent (Fig. 5a). In post-mitotic cells pretreated with Plk1 inhibitor for 4 h (4hBIRO), most cells contained two centriole pairs, each pair containing of one Sas6 signal, associated with the daughter centriole. However, in post-mitotic cells that entered mitosis within 1 h of Plk1 inhibition (1hBIRO), we found a population of centrosomes with closely positioned centriole pairs, but associated with two Sas6 signals of equal or different brightness, indicative of centrioles in reduplication (Fig. 5a). In such centrosomes, a newly accumulated Sas6 signal co-localized with a strong Plk4 signal and with a toroid of Cep152 organized by the mother centriole (Fig. 5b,c). Original daughter centrioles were associated with substantial amounts of gamma tubulin and Cep152, but the two PCMs were not yet separated as analysed by structured illumination microscopy (SIM). Furthermore, the original daughter centrioles were still orthogonal to the mother centrioles. The centres of Sas6 signals belonging to the original daughters were positioned outside the Cep152 toroid organized by the mother centriole. Eventually, additional Sas6 became associated with a weak C1–GFP signal, which corresponded to the short nascent centriole positioned ∼40 nm from the mother centriole's wall, as revealed by electron microscopy (Fig. 5d). Centriole reduplication in 1hBIRO post-mitotic cells strongly supported our hypothesis that centriole block to reduplication was already relieved at the time of mitotic entry, and that it did not require subsequent Plk1-dependent mitotic events.

**Figure 6:**
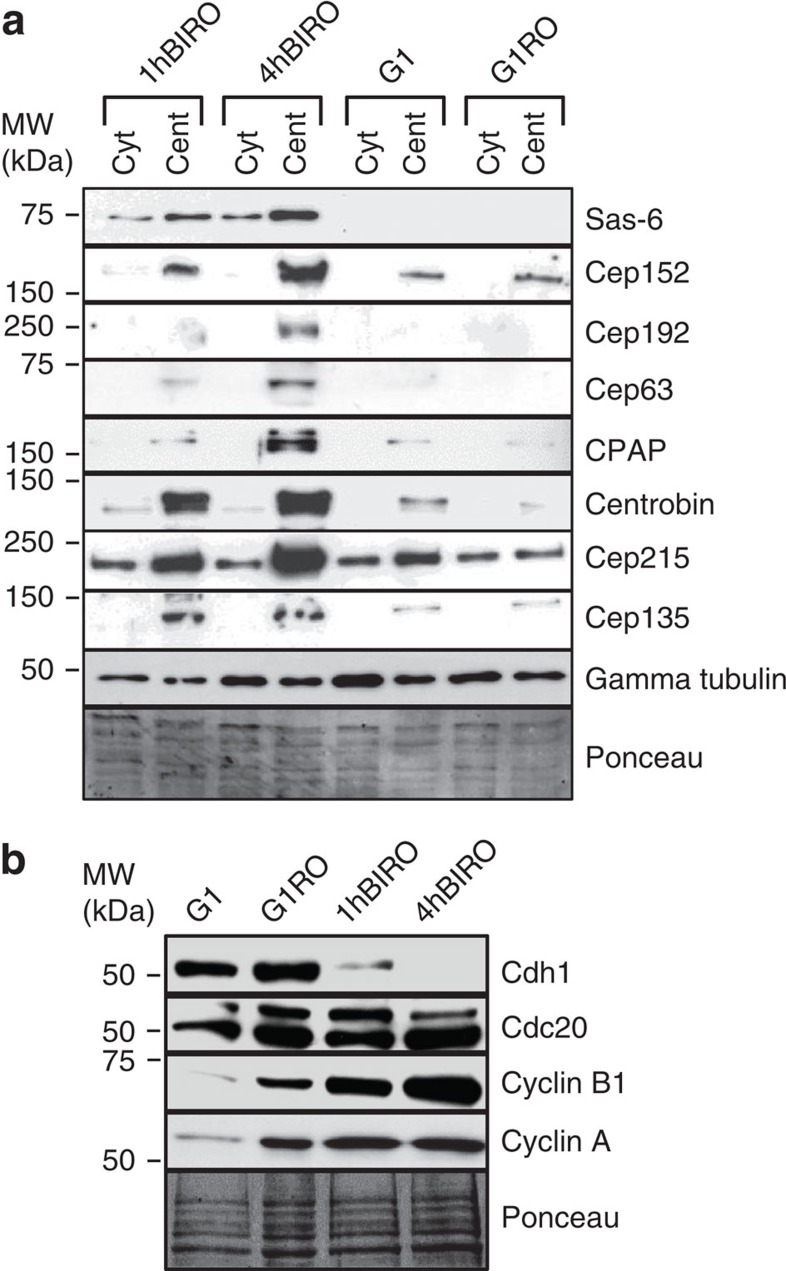
Plk1 inhibition in late G2 prevents proteolytic degradation of centrosomal proteins in post-mitotic cells. (**a**) Western blot analysis of centrosomal proteins under various experimental conditions. Inhibition of Plk1 for 1 h (1hBIRO) or 4 h (4hBIRO) before mitotic entry prevented degradation of centrosomal proteins on the centrosomes. Cent, centrosome-enriched fraction; Cyt, cytosolic fraction. G1=G1 cells, G1RO=post-mitotic cells treated only with RO-3306 (**b**) Western blot analysis of cell cycle regulators from total lysates, under various experimental conditions.

The level of Sas6 is normally low in G1 cells due to its proteolytic degradation^16^, which begins in mitosis in an APC/C^Cdh1^-dependent manner. However, in post-mitotic cells harbouring additional Sas6 foci, neither daughter centriole-associated nor cytosolic Sas6 was degraded (Fig. 6), which was also true for several other centrosomal proteins. Accordingly, Cdh1 was largely reduced in the population of 1hBIRO cells. These interesting data clearly show that APC/C^Cdh1^-dependent degradation of centrosomal proteins was required neither for centriole distancing nor for the loss of centriole block to reduplication. In addition, 1hBIRO cells maintained higher level of cyclins A and B1, which are normally degraded in prometaphase and anaphase, respectively^21^. High levels of centrosomal proteins and cyclins may explain how centriole duplication was supported in post-mitotic cells. These data also argue that degradation of a set of centrosomal proteins during mitosis is Plk1 dependent.

### Centriole distancing does not require centrosome maturation

If centriole distancing was not dependent on proteolytic degradation, we reasoned that it could occur due to the Plk1-dependent accumulation of centrosomal components around the mother centriole. Centrosomal Plk1 activity normally peaks in late G2 inducing a three- to fourfold increase in total centrosome volume due to the accumulation of the PCM components to the outer PCM layer, an event known as centrosome maturation^22^,^23^,^24^. However, expression of Plk1TD in S-phase-arrested or cycling cells outside mitosis does not promote mitotic-like centrosome maturation^5^,^6^ (as illustrated in Figs 1 and 3). Accordingly, depletion of CDK5RAP2/Cep215, a protein that together with Pericentrin regulates the recruitment of PCM components to the mitotic centrosome^25^,^26^, did not change the rate of Plk1-dependent centriole distancing or disengagement (Supplementary Fig. 4). Therefore, mitosis-specific centrosome maturation is not required for centriole distancing.

### Centriole distancing requires CPAP and centrobin

**Figure 7:**
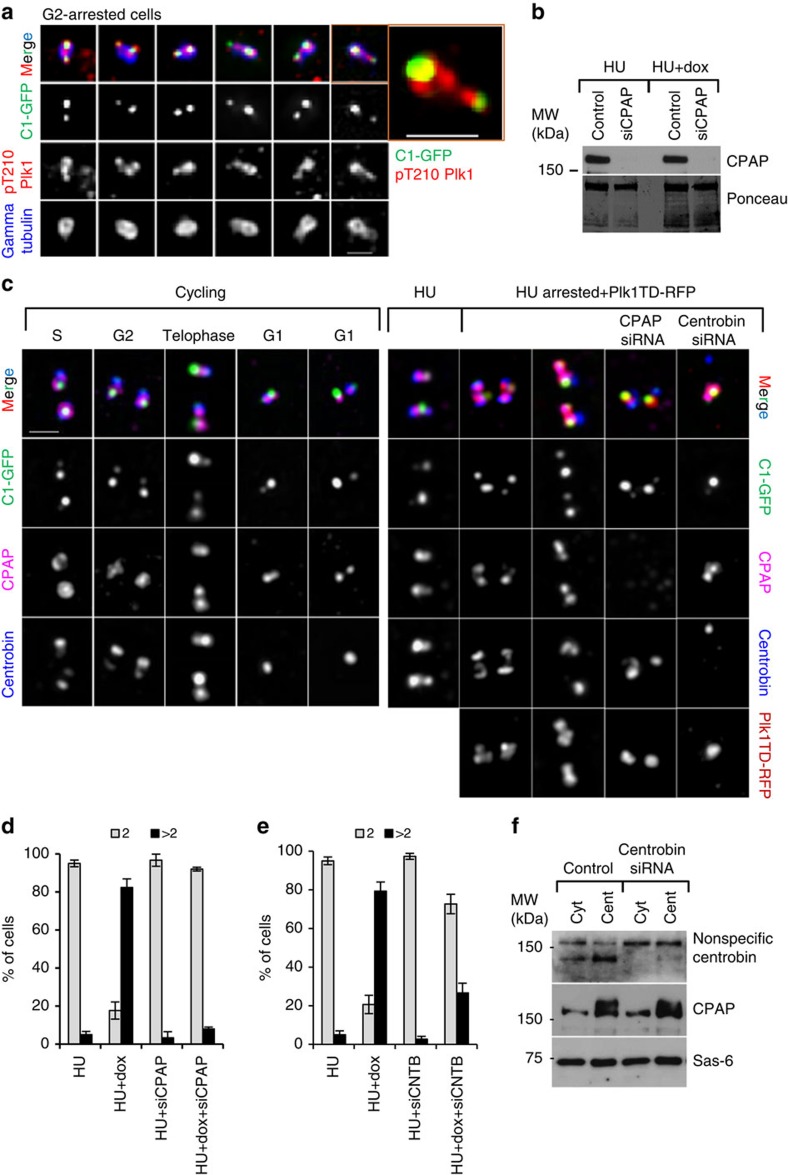
Depletion of CPAP prevents Plk1-dependent centriole distancing and disengagement in S-phase-arrested cells. (**a**) Localization of endogenous active pT210Plk1 on the centrosomes of G2-arrested cells during centriole distancing. Plk1 signal is localized to the site of the mother centriole and along disengaging daughter centrioles. One centrosome from **a** is enlarged to illustrate the details. (**b**) Western blot analysis of total cell lysates from hydroxyurea (HU)-treated cells, to illustrate the typical level of CPAP depletion. (**c**) Analysis of CPAP, centrobin, Plk1TD–RFP and C1–GFP localization, under various experimental conditions. CPAP and centrobin partially co-localize within the centrosomes of cycling cells. Depletion of CPAP or centrobin prevents distancing of the daughter centrioles from the mother centrioles in HU-arrested Plk1TD-expressing cells, as judged by the proximity of their C1–GFP signals. (**d**,**e**) Quantification of individual centrosomes in the cells, judged by the number of resolvable gamma tubulin signals, after CPAP (**d**) and centrobin (**e**) depletion in HU-arrested cells expressing Plk1TD–RFP. Histograms represent the average, and the error bars the s.d. *n*=300, from three independent experiments. (**f**) Western blot analysis of cytosolic and centrosomal fractions from HU-arrested cells, after depletion of centrobin. Centrobin depletion does not change the amount of cytosolic or centrosome-associated CPAP and Sas6. Scale bars, 1 μm.

During centriole disengagement in G2, active endogenous Plk1 closely associates with daughter centrioles (Fig. 7a), and inhibition of Plk1 in S and G2 inhibits daughter centriole elongation, maturation and subsequent disengagement^5^. We thus hypothesized that centriole distancing could occur as a consequence of Plk1-dependent maturation of a daughter centriole. Several centrosomal proteins, including centrosomal protein 4.1-associated protein (CPAP) and centrobin, have been reported to be required for the stabilization and elongation of daughter centrioles^27^,^28^,^29^. Both proteins are associated with daughter centrioles from their formation in the S phase, and through their elongation in G2 and mitosis, and their maturation in G1 (Fig. 7b). To test our hypothesis, we depleted CPAP or centrobin in S-phase-arrested cells after daughter centrioles were already formed, induced Plk1TD expression and monitored the effect of Plk1 expression on centriole distancing and reduplication.

**Figure 8:**
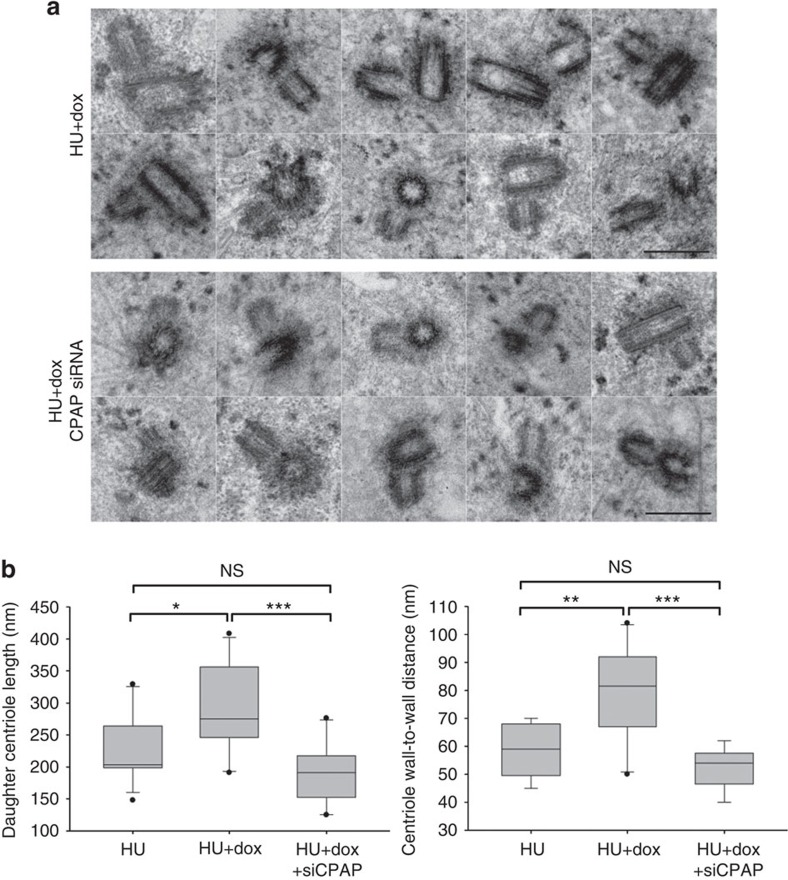
Measurement of centriole distances in Plk1TD-expressing cells depleted for CPAP. Cells were arrested in S phase by hydroxyurea (HU). CPAP was depleted in some samples and expression of Plk1TD–RFP was induced with doxycycline (dox). Cells with duplicated centrioles and centrosome-associated Plk1TD–RFP were visualized on a confocal microscope and fixed for same-cell electron microscopy. (**a**) Examples of centrioles analysed by electron microscopy. One representative section through the centriole pair is presented for each centrosome. Expression of Plk1TD–RFP in control HU-arrested cells leads to centriole distancing and disorientation. Daughter centrioles can be seen in various orientations and at variable distances from the mother centrioles. In CPAP-depleted cells daughter centrioles are short, closely positioned near the wall of the mother centrioles, and orthogonal. (**b**) Quantification of the daughter centriole length and the distances from the mother cariole wall, measured under different experimental conditions from electron micrographs are presented as box and whiskers plots. SigmaPlot software was used to generate the box and whisker plot and shows maximum, minimum, median, upper-quartile and lower-quartile values. The statistical significance between the data sets was determined by a two-tailed *t*-test using Microsoft excel. NS, not statistically different. *P≤0.05, **P≤0.01, ***P≤0.001. Scale bars, 500 nm.

Expression of Plk1TD in S-phase-arrested cells leads to the maturation of daughter centrioles, which is reflected in accumulation of centrosomal proteins such as Cep135 (refs 30, 31), Cep120 (refs 32, 33), hPOC5 (ref. 34) and polyglutamylated tubulin to their sites^5^,^6^. Therefore, we analysed the association of these proteins with daughter centrioles in CPAP- and centrobin-depleted cells, after Plk1 expression. Plk1TD efficiently localized to the centrosomes in depleted cells (Fig. 7b,c,f). However, daughter centrioles stayed in close association with mother centrioles and were associated with a weak C1–GFP signal. Accumulation of centriole maturation factors Cep135, Cep120, hPoc5 and GT335 at the sites of the daughter centrioles did not occur (Supplementary Figs 5 and 6), and centrioles did not reduplicate (Fig. 7d,e). Electron microscopy analysis of the centriole pairs in CPAP-depleted cells expressing Plk1TD were significantly shorter than in control populations and were found closer to the walls of the mother centrioles (Fig. 8). Centrioles in control, undepleted, cells were found in reduplication and/or disoriented.

It has been reported that CPAP accumulation at the site of centriole assembly depends on its interaction with centrobin^35^. However, in our hands centrobin depletion did not reduce overall daughter centriole-associated CPAP levels, as detected by immunofluorescence. Moreover, depletion of centrobin did not change the levels of CPAP in the centrosomal fraction analysed by immunoblotting (Fig. 7f). Likewise, depletion of CPAP did not prevent centrosomal localization of centrobin. The finding that these two proteins could independently localize to the centrioles is in agreement with their only partial co-localization within the centrosome (Fig. 7c). Centrobin localized more distally, towards the C1–GFP signal, while CPAP localized closer to the proximal and middle parts of daughter centrioles. In addition to their partial co-localization on the centrioles, CPAP and centrobin only partially co-localized in the PCM surrounding the mother centriole. Therefore, according to our analysis, localization of these two proteins to the centrosome may not be interdependent.

Depletion of centrobin and CPAP, which are both involved in stabilization of daughter centrioles, prevented Plk1-dependent centriole distancing and disengagement. Overall, these data support our hypothesis that Plk1-dependent maturation of the daughter centriole might be a prerequisite for its distancing from the mother centriole. CPAP, Cep120, Cep135 and centrobin are proposed interacting partners in the context of initiation of centriole assembly, centriole elongation and stabilization. Future studies will need to be oriented towards determining how each of these proteins contributes to centriole distancing.

## Discussion

Disorientation of a daughter and a mother centriole is currently equated with the loss of centriole block to reduplication, and is thought to be a causative effect of the proteolytic events occurring after metaphase to anaphase transition. However, in the presence of active Plk1, centrioles can disengage and reduplicate throughout interphase, thus outside mitosis. To better understand architectural changes occurring within the centrosomes before centriole reduplication, we combined correlative high-resolution light and electron microscopy.

We found that, contrary to current view, orthogonal orientation of the centrioles is not the feature preventing centriole reduplication in human cells. Rather, our data strongly suggest that centriole block to reduplication relies on a close association between a mother and a daughter centriole, which is established at the time of daughter centriole formation, and is relieved in a Plk1-dependent manner both in unperturbed cycling cells and in S-phase-arrested cells expressing active Plk1.

Although the mechanisms leading to the Plk1-dependent loss of association between the mother and daughter centriole are yet to be uncovered, we showed that immature daughter centrioles, in CPAP- or centrobin-depleted cells, cannot respond to Plk1 activity at the centrosome and cannot distance from their mothers to consequently relieve them from duplication block. This finding prompted us to hypothesize that centriole distancing occurs due to gradual Plk1-dependent biochemical maturation of a daughter centriole, which leads to accumulation of PCM components around its proximal parts, stimulating distancing from the mother centriole due to growing spatial restrictions between the two radially organized PCMs.

According to our data, mother centrioles can initiate a new round of centriole duplication if the original daughter centriole distanced to ∼80 nm. By analysing the ultrastructure of centrioles from various cycling mammalian cells by electron microscopy, we demonstrated that the centrioles reach such a distance by prophase, which opens up an exciting possibility that the centriole block to reduplication is relieved already upon mitotic entry, and not after metaphase to anaphase transition, as currently believed.

The final separation of the two centrioles and centriole disorientation, according to our data, is a facultative event to the loss of centriole reduplication block. We speculate that its manifestation could be influenced by various conditions in the cells, such as microtubule dynamics, position of the centrioles in the cell or overall intracellular dynamics.

It has been suggested previously that orthogonally oriented centrioles can duplicate in *Drosophila* wing endocycling cells^36^. Similarly, studies in butterfly primary spermatocytes have indicated that centrioles can duplicate while still engaged^37^. These data presented here strongly corroborate these findings and show that the centriole duplication without centriole disorientation is not a paradox reserved for insect centrioles, but that it also operates in mammalian cells.

## Methods

### Cell culture

HeLa cells expressing C1–GFP^38^ were cultured at 37 °C, 5% CO_2_ in DMEM (Invitrogen), 10% FCS and 1% penicillin/streptomycin. For microscopy analysis, cells were plated on round 170-nm-thick glass coverslips (Warner Instruments). For live-cell analysis, coverslips with cells were mounted in Rose chambers, which were perfused with complete CO_2_-independent medium (Invitrogen). To express Plk1TD–RFP, cells were treated with 1 μg ml^−1^ doxycycline (dox, Sigma).

### Cell synchronization and cell arrest

To avoid potential unspecific effects of the drugs conventionally used for cell synchronization on the centriole cycle, cells were synchronized exclusively by mitotic shake off. Mitotic cells were collected by gently tapping on the tissue culture flasks containing logarithmically growing cells and replated on the coverslip or in the fresh dish, according to the needs of a particular experiment. One hour after replating entire culture was synchronously in G1. These cells were then either cultured without any further treatment and analysed at appropriate time points, or treated with 2 mM HU (Sigma) to induce S-phase arrest, or with 9 μM Cdk1 inhibitor RO-3306 (Tocris Biosciences) to induce G2 arrest^10^. Plk1 inhibitor BI2536 (200 nM)^39^ (Selleckchem) was added to some samples to inhibit endogenous Plk1 activity, when needed. If left untreated, G1 cells progress synchronously through the cell cycle allowing unambiguous determination of the cell cycle phase. Examination of the cells under bright light by differential interference contrast microscopy was used to select cells in a particular phase of mitosis for further imaging or fixation, as illustrated in Supplementary Fig. 7.

### Centriole reduplication assays

Centriole reduplication was promoted either by expression of the constitutively active T210D mutant of Plk1 in cycling or in S-phase-arrested cells, or by arresting cells in G2 using the RO-3306 inhibitor. G2-arrested cells were typically analysed 3–6 h after they reached G2. Cycling cells were typically analysed 16–24 h, and S-phase-arrested cells 8–12 h, after induction of Plk1TD expression.

### Generation of post-mitotic cells with inhibited Plk1

To analyse the effect of Plk1 inhibition on centriole distancing and reduplication in post-mitotic cells, pre-existing mitotic cells were removed from asynchronously growing cells by shake off. The culture was then treated with 200 nM BI2536. Inhibition of Plk1 by BI2536 allows new mitotic entry, but due to the absence of Plk1 activity mitotic cells do not progress beyond prometaphase^39^. Inhibition of Cdk1 with RO-3306 can be then used to induce mitotic exit^8^,^10^. This method was used in this manuscript to generate post-mitotic cells, which entered mitosis within 1–8 h of Plk1 inhibition, depending on a particular experiment. Post-mitotic cells were then fixed for immunofluorescence, or mounted in a Rose chamber for live-cell imaging, where they were directly fixed for correlative electron microscopy analysis. Untreated G1 cells and mitotic cells treated with RO-3306 only were used as controls.

### siRNA transfections

Following short interfering RNA (siRNA) oligonucleotides were used in the study: CPAP: 5′-AGAAUUAGCUCGAAUAGAA-3′, centrobin: 5′-AGUGCCAGACUGCAGCAACTT-3′ and Cep215: 5′-GCAAAGAAGCUACGAGAUU-3′ (Dharmacon Research Inc.). Cells were transfected with 300 nM of siRNA oligonucleotides using Oligofectamine (Invitrogen). To assure complete depletion of the proteins, the siRNA transfection was performed in a 75-cm^2^ cell-culture flask 36 h (for CPAP and centrobin) or 48 h (for Cep215) before the cells were collected by shake off for reduplication experiments. Pre-depleted mitotic cells were then collected and replated. 2 mM of HU was added to the cells 10 h later when centrioles initiated duplication, Plk1TD–RFP was induced by dox in some cultures. At 30 h after the shake off, cells were fixed and the rate of centriole distancing and disengagement was analysed by light or electron microscopy. Using this experimental design the level of targeted proteins already dropped during the first centriole cycle but to the level that still allowed centriole duplication in the second centriole cycle. By the time expression of Plk1 was induced by dox, the levels of depleted proteins further decreased to often undetectable levels on the centrosomes. The efficiency of the depletion was monitored during the experiment by western blot and by immunofluorescence.

### Immunostaining

Cells were fixed with 1.5% formaldehyde in PBS for 5 min and post-fixed with ice-cold methanol for 5 min at −20 °C. After several washes in PBS, the cells were blocked in 1% BSA (Sigma) and 0.05% Tween-20 (Sigma) in PBS for 15–0 min and then incubated with primary antibodies. Alexa Fluor 555-, 405-, 488- or 647-conjugated secondary antibodies (Invitrogen) were used to visualize the proteins. DNA was stained with Hoechst 33342 (Invitrogen). The following primary antibodies were used for immunostaining in this study: mouse monoclonal anti-Sas6 (1:500; sc-81431; Santa Cruz), mouse monoclonal anti-γ-tubulin (1:5,000; T6557; Sigma), mouse anti-centrobin (1:600; ab70448; Abcam), rabbit anti-Cep135 (1:500; ab75005; Abcam), rabbit anti-Cep152 (1:4,000; A302-479 A; Bethyl), rabbit anti-γ-tubulin (1:5,000; T3559; Sigma), rabbit anti-Cep215 (1:2,000; 06-1398; Millipore), goat anti-CPAP (1:500; sc-66747; Santacruz), rabbit anti-CPAP (1:500; 11517-1-AP; Proteintech), mouse anti-GT335 (1:2,000; AG-20B-0020-C100; Adipogen), mouse anti-Plk1(pT210) (1:500, 558400; BD Biosciences) rabbit anti-Plk4 (1:100; kind gift from Dr Kyung S. Lee, NIH Bethesda, USA), rabbit anti-hPOC5 (1:1,000; a kind gift from J. Azimzadeh, Institute Jacques Monod, Paris, France), rabbit anti-Cep120 (1:1,000; a kind gift from Moe R Mahjoub, Washington University in St Louis, USA).

### Immunoblotting

To separate centrosomes from cytosol, cells were washed sequentially with ice-cold 1 × PBS, 8% sucrose in 0.1 × PBS, 8% sucrose in H_2_O, 1 mM Tris pH 8.0+0.46 μl ml^−1^ β-ME and lysed at 4 °C in 1 mM Tris-HCl buffer (pH 8.0) containing 0.5% IGEPAL CA-630 and protease/phosphatase cocktail inhibitors (Roche). The total lysate was then centrifuged at 2,537*g* to remove nuclei. The crude centrosome-containing fraction was separated from the cytosolic fraction by ultracentrifugation of the supernatant at 9,137*g*. Proteins were separated by SDS–polyacrylamide gel electrophoresis, transferred to a polyvinylidene difluoride membrane (162-0255; Bio-Rad) and blocked in 3% milk for 1–2 h, followed by incubation with primary antibody for 1 h at room temperature or overnight at 4 °C. Primary antibodies were used as follows: mouse monoclonal anti-Plk1 (1:500; sc-17783; Santa Cruz), mouse monoclonal anti-Sas6 (1:500; sc-81431; Santa Cruz), mouse monoclonal anti-γ-tubulin (1:10,000; T6557; Sigma), rabbit anti-Cep135 (1:1,000; ab75005; Abcam), rabbit anti-Cep152 (1:4,000; A302-479A; Bethyl), rabbit anti-Cep63 (1:500; 06-1292; Millipore), rabbit anti-Cep215 (1:2,000; 06-1398; Millipore), goat anti-CPAP (1:500; sc-66747; Santa Cruz), mouse anti-Centrobin (1:500; ab70448; Abcam), rabbit anti-Cep192 (1:500; sc-84785; Santa Cruz), mouse anti-Cdh1 (1:200; cc43; Calbiochem), rabbit anti-cyclin A (1:250; D13012_CA; Delta), mouse anti-cyclin B1 (1:2,500; 4135S; Cell Signaling), rabbit anti-Cdc20 (1:5,000; A301-180A; Bethyl). HRP-conjugated secondary antibodies (1:10,000; Amersham) and Clarity western ECL substrate detection kit (170-5060; Bio-Rad) were used to detect the signals. Precision Plus protein standard protein marker (161-0374; Bio-Rad) was used for western blots. Scanned western blot films are provided in Supplementary Figs 11–13.

### Light microscopy

Wide-field images were acquired using a Nikon Eclipse Ti inverted microscope, equipped with a 64-μm-pixel CoolSNAP HQ^2^ camera (Photometrics) and Intensilight C-HGFIE illuminator, using × 100 numerical aperture (NA) 1.42 Plan Apo objective with × 1.5 magnifying tube lens. Two hundred-nanometre-thick Z-sections spanning the entire cell or entire centrosome, as needed, were acquired. AutoQuant X3 software (MediaCybernetics) was used for deconvolution. ImageJ (NIH), and NIS-Elements software package was used to make maximal intensity projections and to assemble acquired images.

For live-cell imaging, cells growing on the coverslip were assembled in a Rose chamber and imaged using a Nikon Eclipse Ti inverted microscope equipped with × 100 and × 60 NA 1.45 Plan Apo objectives, Yokogawa spinning disc, 405, 488, 561 and 640 nm laser launch (Agilent Technology MCL-400), back-illuminated 16-μm-pixel or 13-μm EMCCD camera (Andor, DU897 and DU888, respectively), × 100 NA 1.42 Plan Apo objective lens with × 1.5 magnifying tube lens and a × 1.2 or × 2 relay lens.

SIM was performed on N-SIM, Nikon Inc., equipped with Apo TIRF × 100 NA 1.49 Plan Apo oil objective, 405, 488, 561 and 640 nm excitation lasers and a back-illuminated 16-μm-pixel EMCCD camera (Andor, DU897). Z-Sections with a Z-distance of 100 nm spanning the entire volume of the centrosomes were acquired in 3D SIM mode generating 15 images per plane (five phases, three angles) as a raw image, which was further computationally reconstructed to generate a super-resolution image. After image reconstruction, colour channels were corrected for XYZ shift, using a macro written in NIS-Elements^5^, and the signals of 100-nm multi-spectral fluorescent spheres (TetraSpeck beads, Invitrogen) included in the mounting medium. For surface rendering, 3D visualization of the centrosomes and various measurements, we used NIS-elements software package. To assure proper comparison of signal intensities, cells were imaged using identical imaging settings across all samples in an individual experiment. For a given antibody staining or expressed protein, all images were processed identically during figure assembly. The levels of C1–GFP were sometimes differentially adjusted between the panels of one figure, only to unambiguously illustrate the position of the dimmer daughter centrioles within the centrosome.

### Measurement of C1–GFP signal distances

For time lapse, a Z-stack spanning entire centrosomes was recorded every 10 s during at least 5 min. The *X*, *Y* and *Z* coordinates of centres of C1–GFP signals were then manually extracted for each time point from *XY*, *XZ* and *YZ* projections in ImageJ (Supplementary Fig. 9). Extracted *X*, *Y* and *Z* coordinates were then used to calculate the distances between two signals in 3D volume, using the 3D Pythagorean theorem. The voxel size was from 88 × 88 × 200 to 72 × 72 × 200 nm for time-lapse recordings (depending on which combination of lens and charge-coupled device camera was used), 43 × 43 × 200 nm for wide-field imaging and 31 × 31 × 100 nm for reconstructed SIM images. The same principle was used to measure the distances between C1–GFP signals from fixed cells, or between any two dot-like signals within the corrected multicolour image.

### Correlative light and electron microscopy (CLEM)

A cell of interest was first analysed using a confocal microscope and then fixed with 2.5% glutaraldehyde. Two hundred-nanometre-thick Z-sections through the entire cell volume were recorded to register the position of the centrioles within the cell. The position of the cell on the coverslip was marked with a diamond objective scribe. The cells were prepared for electron microscopy as described earlier^5^. In brief, cells on the coverslip were washed in PBS, pre-stained with osmium tetroxide and uranyl acetate, and embedded in EMbed-812 resin. Eighty-nanometre-thick serial sections were then sectioned, stained with uranyl acetate and lead citrate, and imaged using a transmission electron microscope (Hitachi) operating at 80 kV. Image analysis and alignment of the serial sections were performed in ImageJ and Photoshop, respectively.

### Measurement of centriole length in cycling cells

To accurately determine centriole length from electron micrographs we applied rigorous criteria. First, a complete serial section set was obtained for almost all centrioles measured in the study. Second, if a complete set of serial sections was not obtained, centriole length was measured only if the centriole was sectioned longitudinally (longitudinally sectioned centrioles span through maximally four consecutive sections, two of which are needed to unambiguously determine longitudinal orientation). Obliquely sectioned mother centrioles were measured only if they were completely sectioned within five consecutive sections. This criterion assured that a 400–500-nm-long centriole is not tilted more than 10 or 20 degrees, respectively, to the sectioning plane (Supplementary Fig. 9) and guaranteed the measuring error ≤5% for the centrioles tilted ≤10°, and ≤9%, for the centrioles tilted 10–20°. Note that most measured centrioles were completely sectioned within four consecutive sections. Since daughter centrioles in cycling cells could be of various lengths, only longitudinally sectioned daughter centrioles from cycling cells were measured and included in statistics. To measure the length of the centriole, a 200-nm-wide rectangular box was aligned along the wall of the centriole in the central serial section, and the length of the box was measured (Supplementary Figs 9 and 10).

### Measurement of centriole wall-to-wall distance

The distance between the walls of the mother centriole and the proximal end of the daughter centriole, during cell cycle and in S-phase-arrested cells (used in Fig. 4) were measured from electron micrographs. Only centriole pairs with the daughter centrioles sectioned longitudinally were measured. A 200-nm-wide rectangular box was aligned along the wall of the daughter centriole in the central serial section, and the distance between the centre of proximal end of the box and the closest microtubule (microtubule C) belonging to the mother centriole was determined (Supplementary Fig. 10).

### Determination of spatial arrangement between the centrioles

Centriole wall-to-wall distance and spatial arrangement between the centrioles were measured in G2-arrested cells from electron micrographs. Stringent criteria applied for the measurement of the length of the mother centrioles in cycling cells were also used here (Supplementary Figs 9 and 10). Angle between the centrioles was assessed from longitudinally sectioned centrioles, as illustrated in Supplementary Fig. 11. The angle between the centrioles was measured directly from the central serial section. The lines through the centre of the centriole cylinders were drawn and the angle between the two lines was determined. If one of the centrioles was in cross-section, the distance between the line drawn through the longitudinally sectioned centriole cylinder and the centre of the cross-sectioned centriole was used to assess the lateral shift between the axes of the two centrioles. However, only the measurements obtained from longitudinally sectioned centrioles were included in final statistics.

### Statistical analysis

The statistical significance between two data sets was determined by a two-tailed *t*-test using two samples for means for paired samples or two samples assuming unequal variance for unpaired samples, in Microsoft Excel. NS, not significantly different, *P≤0.05, **P≤0.01, ***P≤0.001. SigmaPlot software was used to generate the Box and whisker plot, which shows maximum, minimum, median, upper-quartile and lower-quartile values.

## Additional information

**How to cite this article**: Shukla, A. *et al.* Plk1 relieves centriole block to reduplication by promoting daughter centriole maturation. *Nat. Commun.* 6:8077 doi: 10.1038/ncomms9077 (2015).

## Supplementary Material

Supplementary Information Supplementary Figures 1-14
