# Supplementary material for: Plk1 relieves centriole block to reduplication by promoting daughter centriole maturation
Source: Nat Commun. 2015 Aug 21;6:8077. doi: 10.1038/ncomms9077 (PMC4560806; doi:10.1038/ncomms9077)
Supplement: Supplementary Information — Supplementary Figures 1-14 [file ncomms9077-s1.pdf]

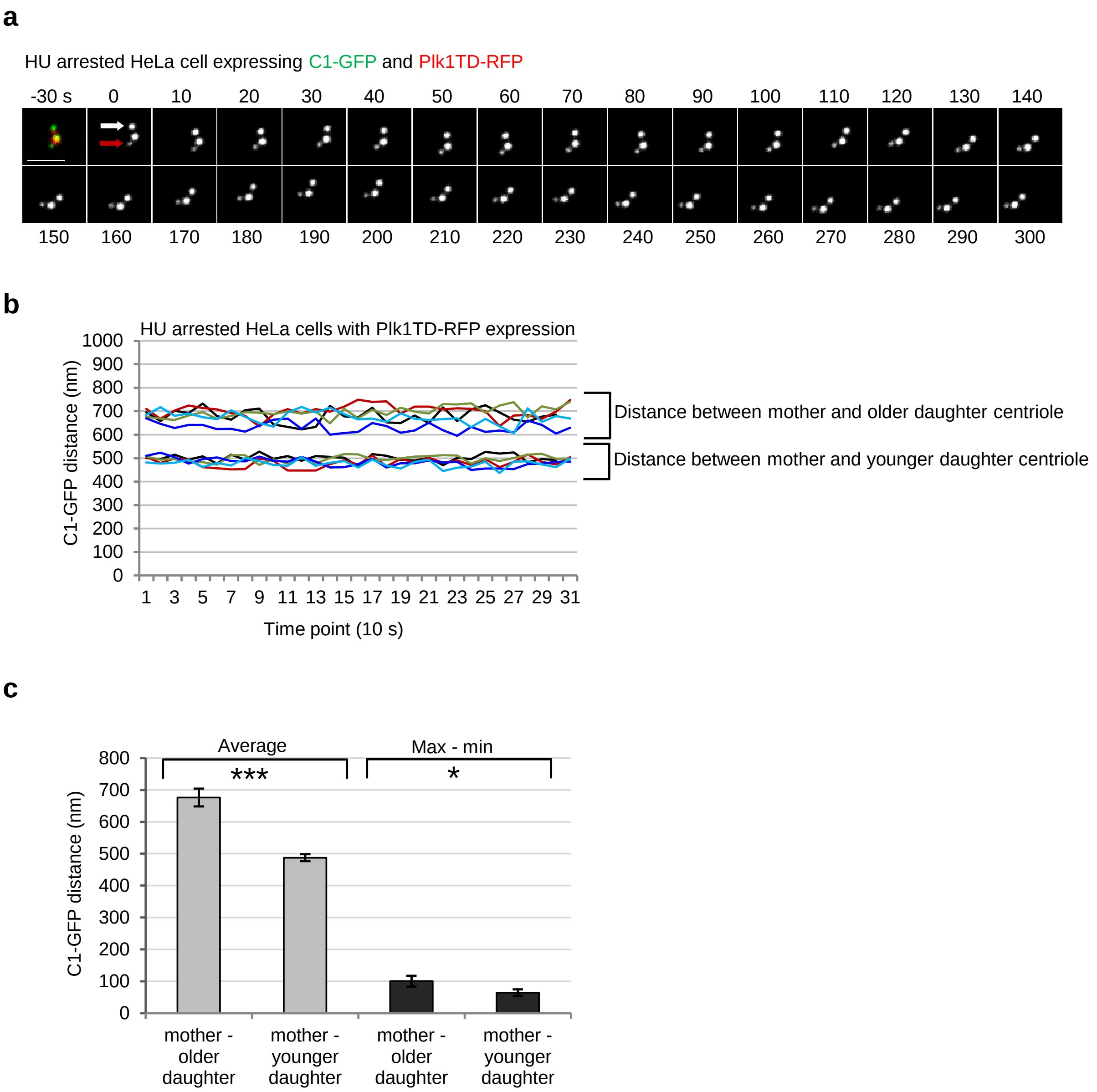

Supplementary Figure 1. Mother centrioles can reduplicate while in the close association with original daughter centriole. (**a**) Stills from a 5 min time lapse recorded with 10 s resolution. The original daughter centriole (white arrow) and a new daughter centriole (red arrow) are associated with the mother centriole as it moves in the cell. (**b, c**) Analysis of C1-GFP distances from time lapse recordings as presented in (**a**). Distances between mother and daughter associated C1-GFP signals were calculated and plotted. n=5 centrosomes, 31 time points per centrosome. Histogram represents the average C1-GFP distance and the average

difference between maximal and minimal C1-GFP distance from five time-lapse recordings. The error bars are the standard deviation. The statistical significance between the data sets was determined by two-tailed t-test using Microsoft excel, NS=not statistically different, $^*P \leq 0.05$, $^{***}P < 0.001$.

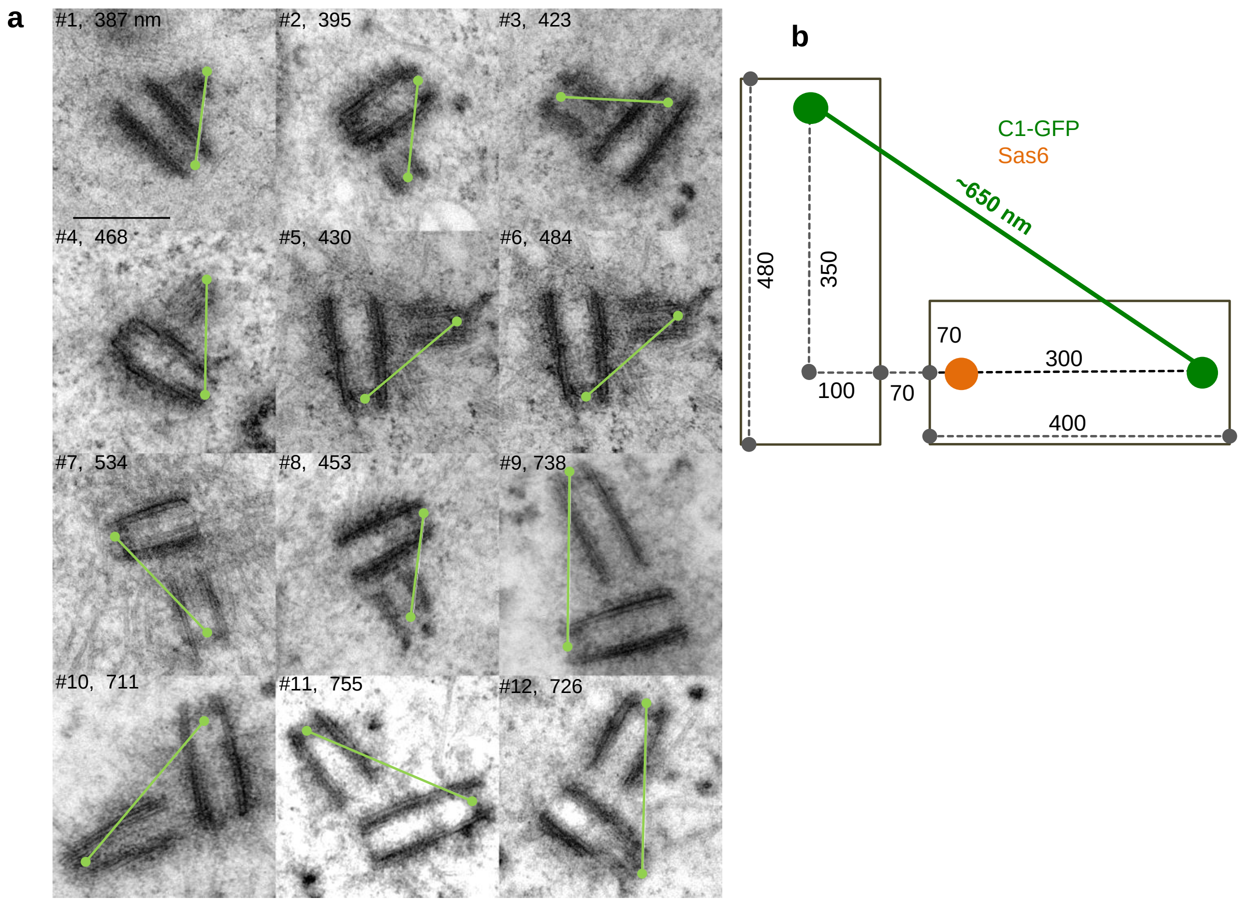

Supplementary Figure 2. Centrin1-GFP is localized ~30 nm from the distal edge of the centrioles. (**a**) To correlate the position of the C1-GFP signals within the centrioles the distances between mother and daughter centriole C1-GFP signals were first determined by light microscopy from 3D recordings obtained from fixed samples. These values (in nm) are presented for each centriole pair. The same centriole pairs were then analyzed by electron microscopy. The line, the length of which corresponded to the measured C1-GFP value, was then superimposed on the middle of both centriole cylinders, at even distance from each distal end. The tips of the line corresponded to the probable C1-GFP position within the centriole. The average distance from the end of the line and the end of the centriole cylinder was 34.2±21 nm, n=12 centriole pairs. All 12 centriole pairs that were measured were sectioned longitudinally or near longitudinally. Scale bar: 400 nm. A central serial section is presented for each centriole pair. (**b**) The maximal theoretical distance between the distal parts of two orthogonal centrioles

of HeLa cells during G2 arrest is 650 nm. This calculation is based on the measurements of the centrioles obtained from electron micrographs as follows: the average length of mother and centriole in G2-arrested cells is 484±49 (n=12) and 408±43 (n=11) nm, respectively. The distance between the walls of the centrioles in G2 cells is 70 nm, the average centriole diameter is ~200 nm. The position of C1-GFP signals is 30 nm from the distal end of the centriole. Cartwheel protein Sas6 (orange dot) is indicated in the middle of a cartwheel ~70 nm from the proximal part of the daughter centriole. Please note that some of the centriole pairs presented in this figure are presented elsewhere in the manuscript to illustrate some other aspect of centriole architecture.

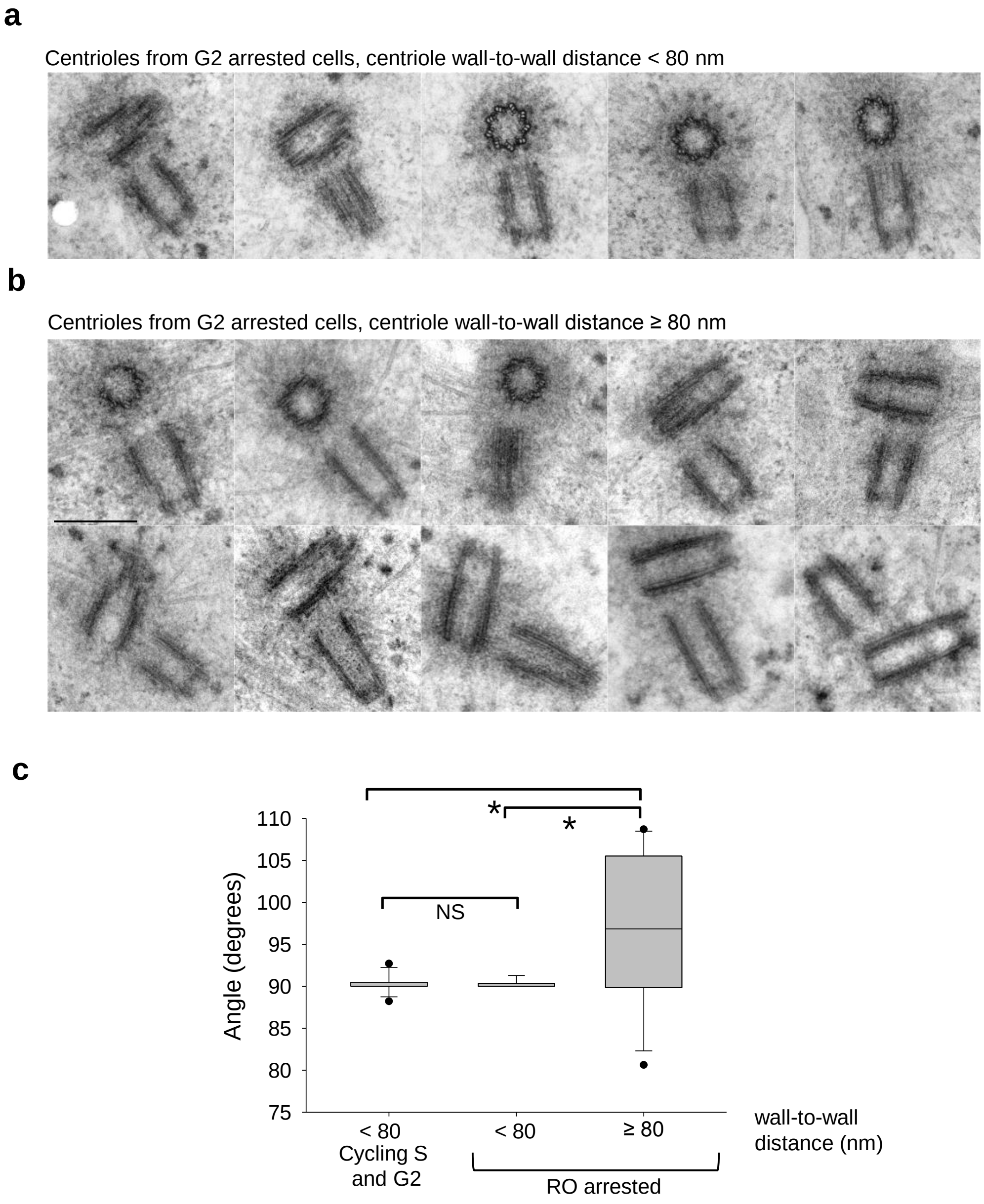

Supplementary Figure 3. Centrioles distanced to 80 nm begin to lose orthogonal orientation. Examples of the mother-daughter centriole pairs from G2 arrested cells. A central serial section is presented for each centriole pair. (**a**) Centriole pairs with the centriole wall to wall distance <80 nm were found in orthogonal orientation. (**b**) The centrioles at the distance ≥80 nm frequently lost orthogonal orientation and/or were shifted along the wall of the mother centriole. (**c**) Angles between the mother and daughter centrioles from cycling interphase cells and G2 arrested cells were measured as explained in Methods and presented as a Box and

whiskers plot. SigmaPlot software was used to generate the Box and whisker plot shows maximum, minimum, median, upper quartile and lower quartile values. The statistical significance between the data sets was determined by two-tailed t-test using Microsoft excel, NS=not statistically different, * $P \leq 0.05$. Please note that some of centriole pairs shown in this figure are presented elsewhere in the manuscript to illustrate some other aspect of centriole architecture.

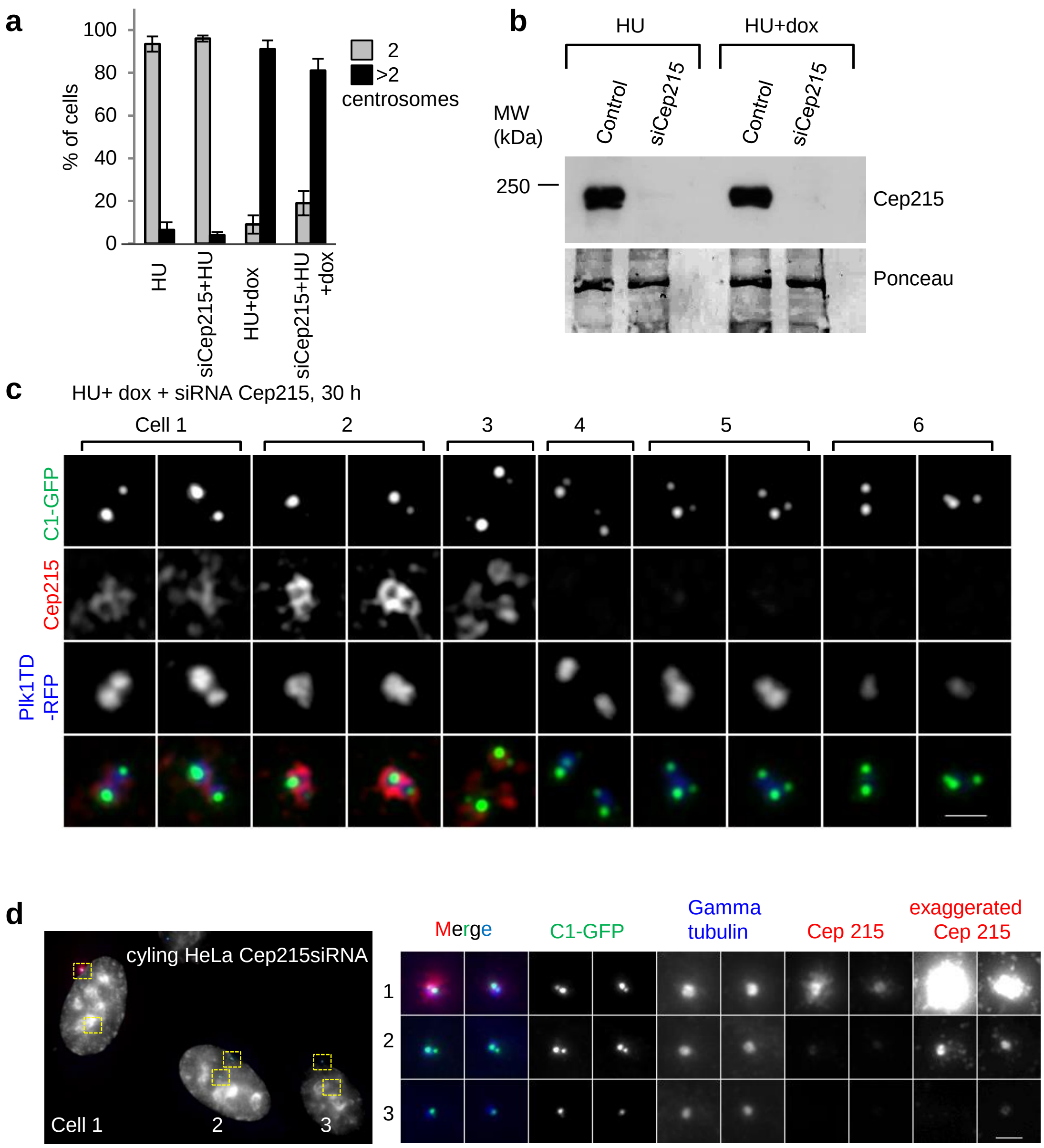

Supplementary Figure 4. Depletion of Cep215 does not affect centriole engagement status in cycling or S phase arrested HeLa cells. (**a**) Quantification of centrosome numbers under various experimental conditions. The number of individual Gamma tubulin signals was counted. Histogram represents the average and error bars the standard deviation. n=300, from three independent experiments. (**b**) Western blot analysis of total cell lysates from Hydroxyurea (HU) treated cells without or with (dox) Plk1TD-RFP expression, to illustrate typical level of Cep215 depletion. (**c**) Example of six independent cells from the same coverslip treated with

siRNA for Cep215, to illustrate various levels of Cep215 associated with the centrosomes. Judged by the distance and the number of C1-GFP signals, Plk1TD-RFP promotes centriole distancing and reduplication, irrespective of the level of Cep215 associated with the centrosomes. (**d**) Cycling cells were transfected with Cep215 siRNA on the first and the third day of the experiment. On day six, the cells were fixed and labeled for Cep215. The panel illustrates three adjacent cells with different levels of Cep215. Cep215 depletion diminished the amount of gamma tubulin associated with the centrosomes (cell 2 and 3). Resident centrioles in cells with undetectable Cep215 were occasionally found either unduplicated or associated with a weak C1-GFP signal (illustrated by cell 3). Intensity of Cep215 was exaggerated to better illustrate the range of centrosome-associated Cep215 level among the cells. Scale bars: 1 μm.

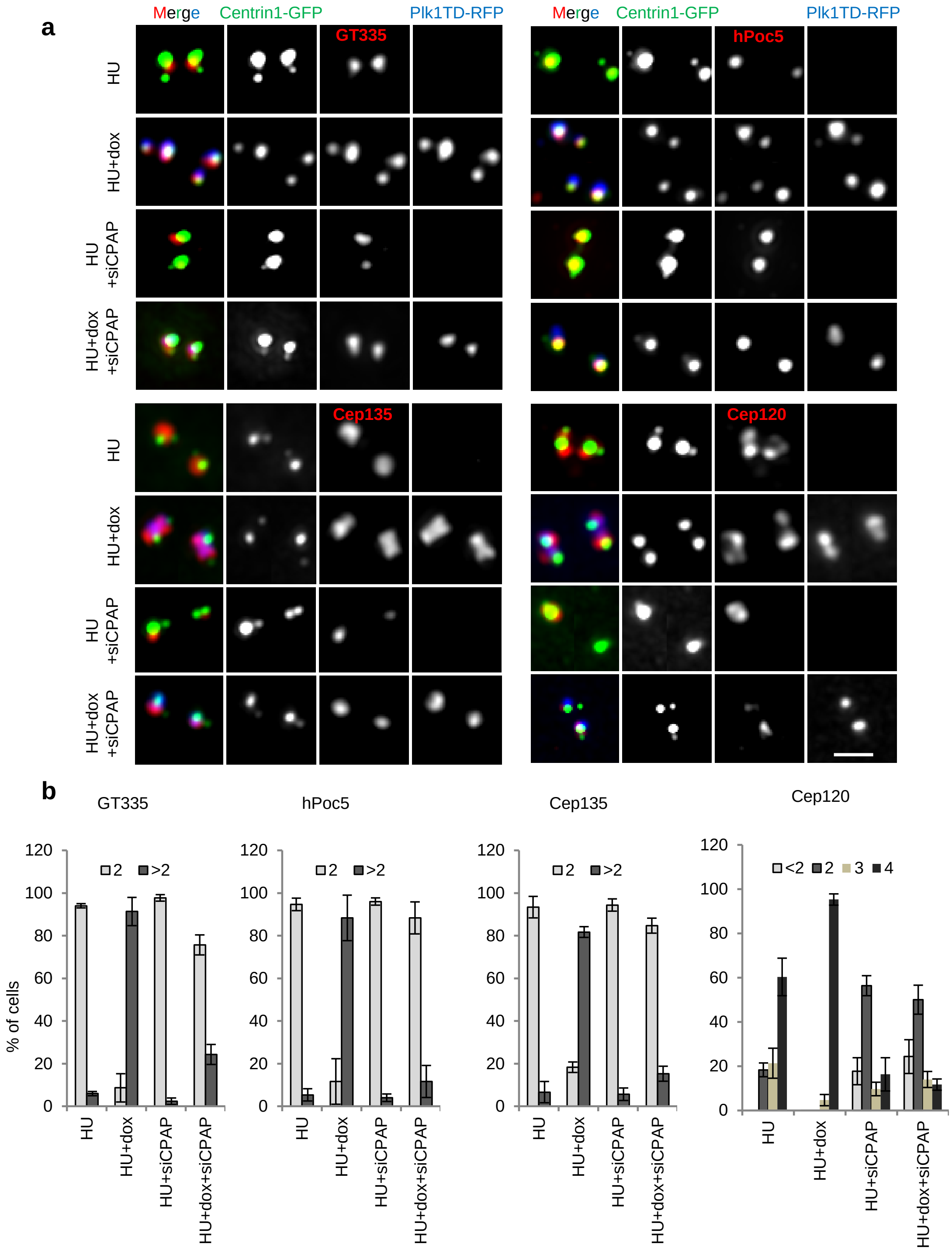

Supplementary Figure 5. CPAP depletion prevents Plk1-dependent accumulation of maturation markers on the daughter centrioles. S phase arrested cells were depleted from CPAP and expression of Plk1TD-RFP was induced by doxycycline. (**a**) The effect of CPAP depletion on accumulation of hPOC5, Cep120, Cep135 and polyglutamylated tubulin (detected

by GT335 antibody) to the sites of the daughter centrioles was analyzed by immunofluorescence. (**b**) Quantification of independent signals for noted centrosomal proteins. Histogram represents the average and the error bars the standard deviation. n=300, from three independent experiments. Scale bar: 1 μm.

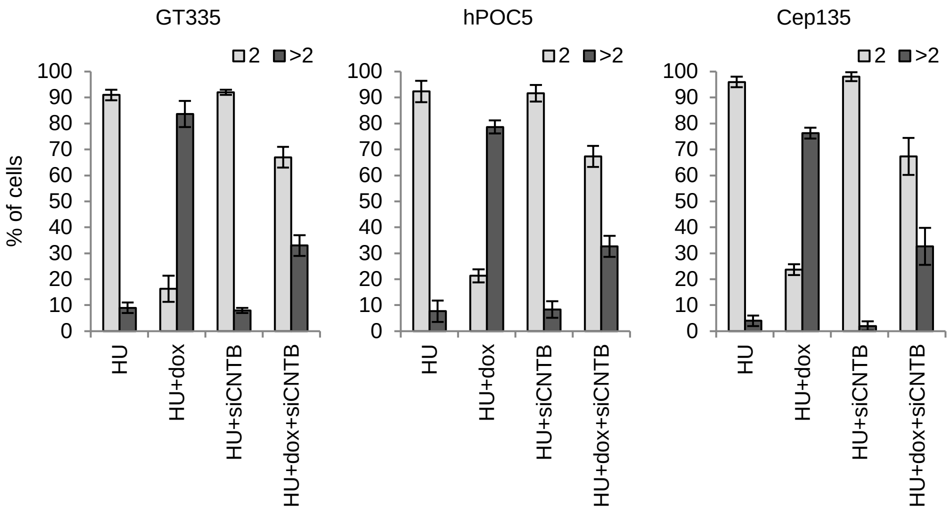

Supplementary Figure 6. Depletion of Centrobin prevents Plk1-dependent centriole distancing and accumulation of maturation markers on the daughter centrioles. S phase arrested cells were depleted from Centrobin and expression of Plk1TD-RFP was induced by doxycycline. The effect of Centrobin depletion on accumulation of hPOC5, Cep120, Cep135 and polyglutamylated tubulin (detected by GT335 antibody) to the sites of the daughter centrioles was analyzed by immunofluorescence. Histograms present quantification of independent immunofluorescence signals for noted centrosomal proteins. Values are the average and the error bars are the standard deviation. n=300, from three independent experiments.

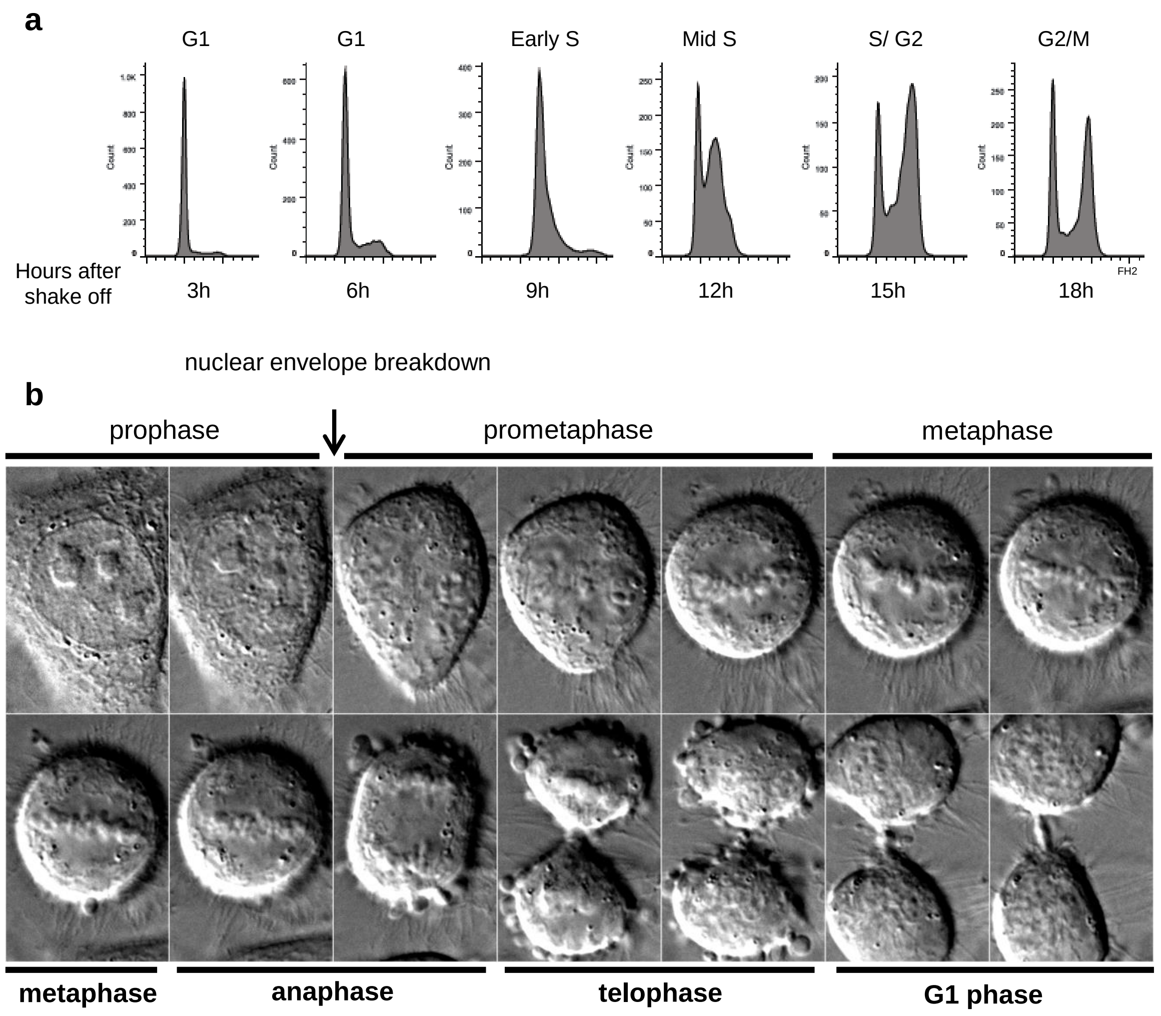

Supplementary Figure 7. Cell synchronization by mitotic shake off and determination of the mitotic phase. (**a**) FACS analysis of cell population synchronized by shake off, 3, 6, 9, 12, 15, and 18 h after the shake off. Mitotic HeLa cells were collected by gently tapping on the culture flask containing logarithmically growing cells and re-plated. If untreated, G1 cells progress synchronously through the cell cycle and by 18 h most cells reach G2 phase. (**b**) Examination of cells by Differential Interference Contrast (DIC) microscopy can be used to unambiguously identify cells in needed stage of mitosis for further filming or fixation.

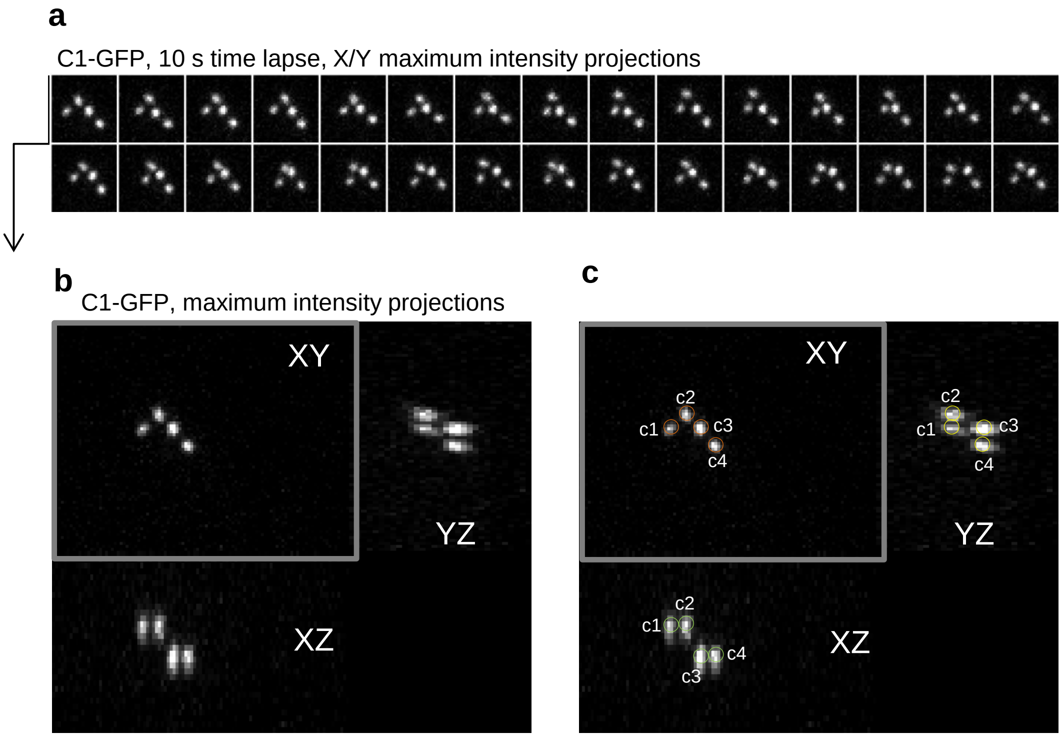

Supplementary Figure 8. Measurement of Centrin1-GFP signal distances from time lapse recordings. To determine distances between two C1-GFP signals in live cells, a Z stack spanning entire centrosomes is first recorded every 10 s during 5 min. (**a**) A series of time frames from one such recording is presented. Four C1-GFP signals belong to four centrioles. XY, XZ and YZ projections are presented in (**b**). Coordinates of centers of each spot is then manually determined from XY and either XZ or YZ projection, in ImageJ (**c**). 3D Pythagorean Theorem is then used to calculate the distances between two signals in 3D volume, taking in account the size of the voxel. Determining coordinates of individual signal requires that the signals do not overlap in two orthogonal projections. This condition is almost always satisfied in our recordings.

a

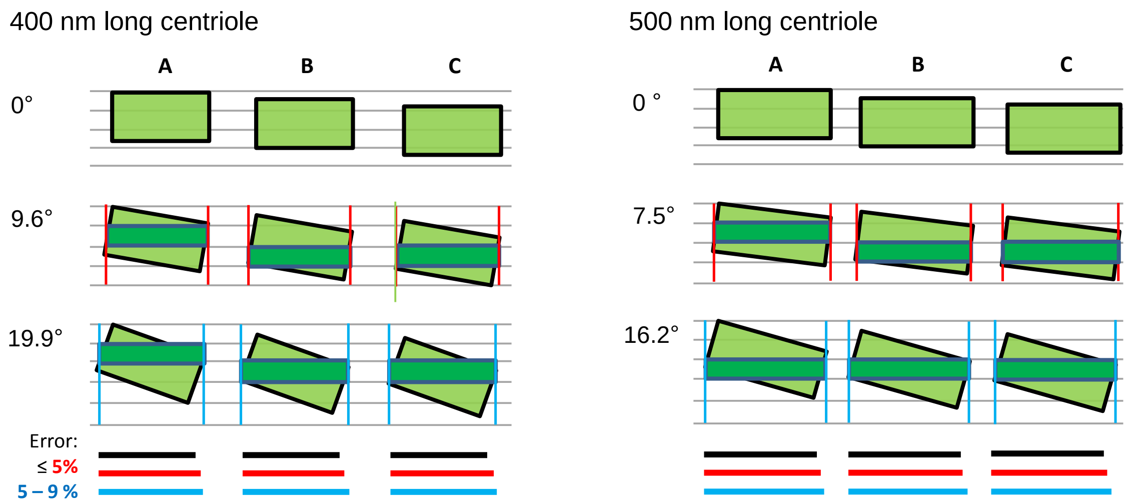

b

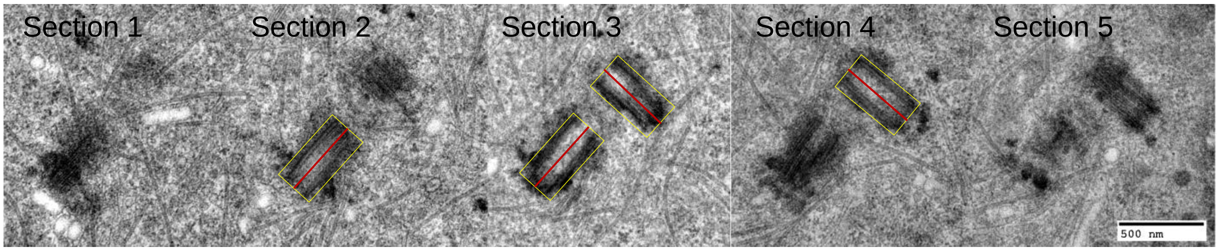

Supplementary Figure 9. Measurement of centriole length from electron micrographs. (**a**) Schematics illustrating possible positions of a 400 or a 500 nm long centriole, with respect to the thickness of the 80 nm serial section (gray horizontal lines). Centrioles sectioned within three (A), four (B), or five (C) consecutive sections are illustrated. Maximal sectioning angle with respect to the sectioning plane for a centriole sectioned within 4 or 5 consecutive sections is ≤ 10º or 10–20º, respectively . Images of the centrioles obtained by electron microscope are the projections of a 80 nm thick section. Therefore, oblique sectioning through the centriole can contribute to the measuring error of the centriole length from electron micrographs. Using standard trigonometry formulas we calculated maximal measuring error for the centriole configurations presented for nine illustrated positions of the centrioles. We found that maximal measuring error for the centrioles sectioned within four sections (therefore tilted 0-10º) is 5%, and for the centrioles sectioned within five sections 5 - 9%. Black numbers represent the maximal tilt of the centrioles in degrees. The projected length of the centriole is illustrated as a

comparison between the length of the black, red and blue line. Calculated maximal error is indicated in %. (**b**) Example of two independent centrioles from cycling G1 cell, sectioned through four consecutive 80 nm thick sections. To measure the length of the centrioles a 200 nm wide rectangular box was aligned along the wall of the centrioles, and the length of the box was measured. The length of both centrioles can be measured from two central sections with an estimated error of ≤ 5%.

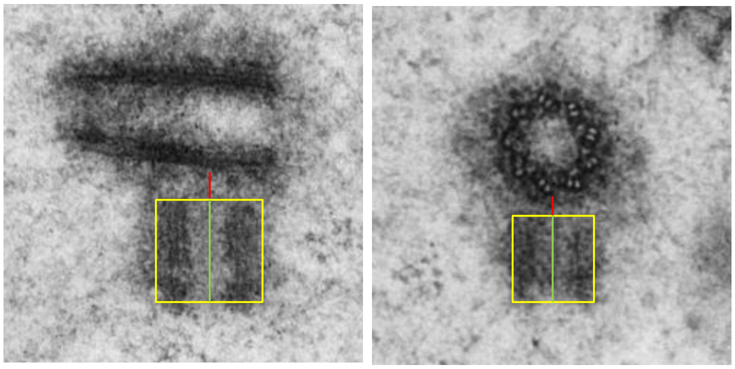

Supplementary Figure 10. Measurement of the centriole wall to wall distances and daughter centriole length in cycling cells from electron micrographs. A 200 nm wide rectangular box (yellow) was aligned along the wall of the daughter centriole in the central serial section and the distance between the center of proximal end of the box and the closest microtubule (microtubule C) belonging to the mother centriole was measured (red line). The length of the box was measured to determine the length of the daughter centriole (green line).

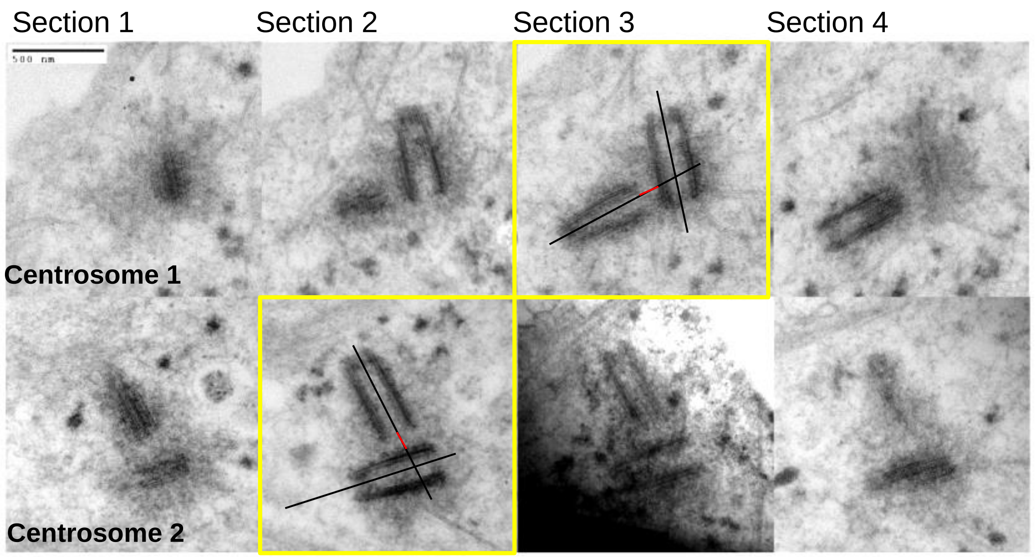

Supplementary Figure 11. Analysis of spatial arrangement of the centrioles in G2-arrested cells from electron micrographs. Two centriole pairs sectioned longitudinally are presented. Angle between two centrioles was measured directly from the central serial section (outlined by a yellow square). The lines through the center of the centriole cylinders were drawn and the angle between the two lines was measured. The distance between the proximal end of the daughter centriole and outer microtubule belonging to the mother centriole was measured (red line) and represented wall to wall centriole distance.

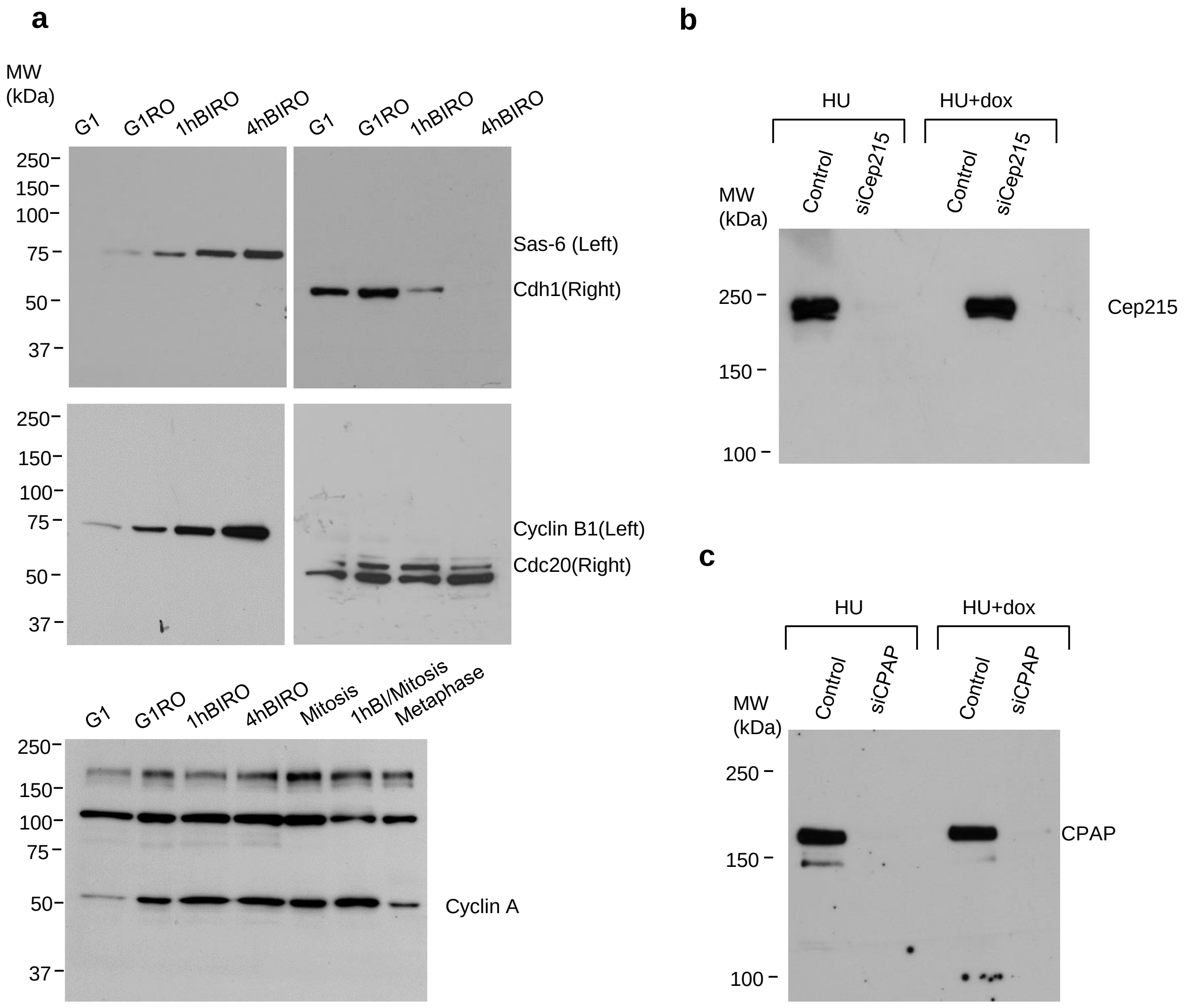

Supplementary Figure 12. Scanned western blot films used in **(a)** Fig. 6c**, (b)** Fig 5c**,** and **(c)** Fig. 7d**.**

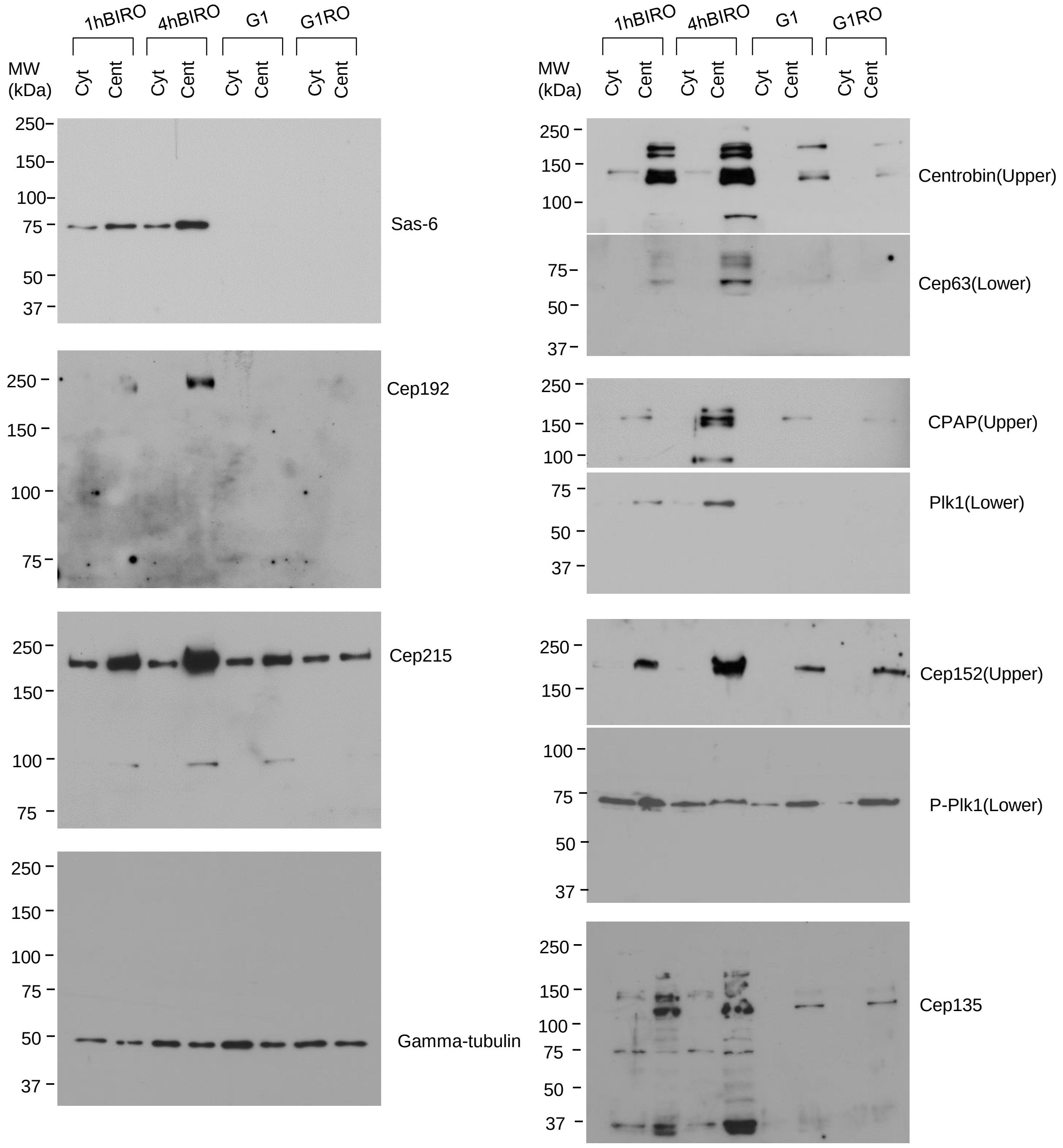

Supplementary Figure 13. Scanned western blot films used in Fig 6b.

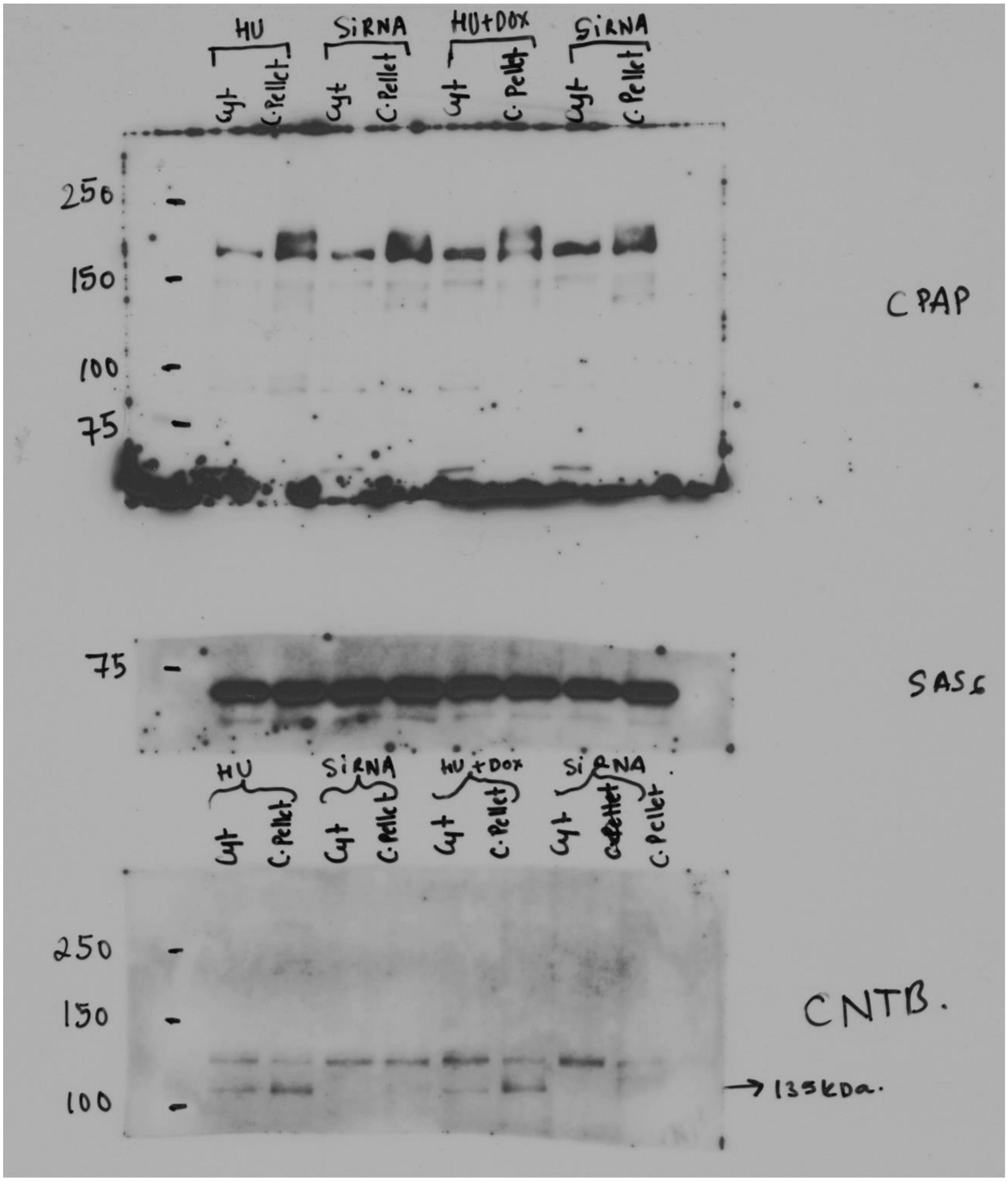

Supplementary Figure 14. Scanned western blot films used in Fig 7.
